# Integrating spatial indicators in the surveillance of exploited marine ecosystems

**DOI:** 10.1371/journal.pone.0207538

**Published:** 2018-11-21

**Authors:** Marta Mega Rufino, Nicolas Bez, Anik Brind’Amour

**Affiliations:** 1 IFREMER—Centre Atlantique, French Research Institute for Exploitation of the Sea, Département Ecologie et Modèles pour l'Halieutique (EMH), France; 2 Centro de Ciências do Mar (CCMAR), Universidade do Algarve, Campus de Gambelas, Faro, Portugal; 3 MARBEC, IRD, Univ Montpellier, CNRS, Ifremer, Sète, France; Swedish University of Agricultural Sciences and Swedish Institute for the Marine Environment, University of Gothenburg, SWEDEN

## Abstract

Spatial indicators are used to quantify the state of species and ecosystem status, that is the impacts of climate and anthropogenic changes, as well as to comprehend species ecology. These metrics are thus, determinant to the stakeholder’s decisions on the conservation measures to be implemented. A detailed review of the literature (55 papers) showed that 18 spatial indicators were commonly used in marine ecology. Those indicators were than characterized and studied in detail, based on its application to empirical data (a time series of 35 marine species spatial distributions, sampled either with a random stratified survey or a regular transects surveys). The results suggest that the indicators can be grouped into three classes, that summarize the way the individuals occupy space: occupancy (the area occupied by a species), aggregation (spreading or concentration of species biomass) and quantity dependent (indicators correlated with biomass), whether these are spatially explicit (include the geographic coordinates, e.g. center of gravity) or not. Indicator’s temporal variability was lower than between species variability and no clear effect was observed in relation to sampling design. Species were then classified accordingly to their indicators. One indicator was selected from each of the three categories of indicators, to represent the main axes of species spatial behavior and to interpret them in terms of occupancy-aggregation-quantity relationships. All species considered were then classified according to their relationships among those three axes, into species that under increasing abundancy, primarily increase occupancy or aggregation or both. We suggest to use these relationships along the three-axes as surveillance diagrams to follow the yearly evolution of species distributional patterns in the future.

## Introduction

The ecological state of a species is reflected on its abundance, which in turn is related with the available space where the proper conditions are met, whether these are environmental, inter and intra-specific (MacCAll’s bassin hypothesis, [1]). Changes in species spatial distributions can be used as a proxy of the ecological state of species and ecosystems as it reflects species response to current major challenges, such as climate change or anthropogenic impacts. Species spatio-temporal dynamics are summarized by spatial indicators, which can then be used by decision makers for management and conservation. When relevant spatial distribution information is incorporated into management, risks and uncertainties can be strongly reduced [2,3]. However, spatial indicators are not typically used in management as they should [2–5].

Further, regulations imposed by management generally have a spatial component either explicitly through time and area closures, or implicitly through allocation of quota to regions or to fleet sectors with different geographical distributions. The European Union recently adopted the Marine Strategy Framework Directive (2008/56/EC and 2017/848/EU) with the objective of reaching a Good Environmental Status in all European waters, by 2020. The implementation of the MSFD relies on indicators to monitor the state of species and ecosystems status [6,7], whose values will determine the management actions to be taken. In particular, changes of species geographical distributions are monitored using spatial indicators, which quantify the species distributional range and, where relevant, its pattern [6–8]. The current work was developed within the framework of the selection of spatial indicators to be used within the MSFD.

In the early 2000's large efforts were devoted to developing spatial indicators useful to fishery management [9–12]. Since then, spatial indicators have been widely used in different studies [13–20], but their properties, limitations and intrinsic relationships have hardly been tested [21,22]. Previous works using spatial indicators used for monitoring marine species have been briefly reviewed in the current work and some particularities of those indicators, were tested and discussed.

Spatial indicators are often used as an ensemble, as it is generally accepted that ecological assessment must be done by integrating several indicators [12,22–24]. Building a “dashboard” of indicators increases the opportunities of picking up changes in critical factors over time. Still, the use of several correlated indicators can raise important issues of redundancy and collinearity. A possible solution is to use the orthogonal axes resultant of a multivariate analysis applied to the indicators results [25], but other solutions have been also proposed, like selecting the most temporally continuous indicators using min/max autocorrelation factors (MAFA) [26–28]. However, such approaches have the disadvantages of only summarizing/decomposing the set of indicators in principal components, potentially causing bias due to the analytical procedures as well as losing the sense or the scale of the indicators per se. As the number of indicators increases, so their usefulness diminishes and the risk of having inconsistency between results increases [29], thus it becomes difficult to match adequate management measures. Also, there is a need for rigorous selection procedure to identify the minimum number of indicators necessary to support management [7]. Therefore, the number and identity of spatial indicators remain an important issue that is still under debate. There is an urge for the conservation community to set a rigorous framework for defining spatial indicators [30], for which it is essential to study their limitations and behavior.

On the other hand, the ecological concepts behind the indicators have been widely debated, although generally apart from management and conservation. As stated before, spatial indicators are used to quantify the way the species occupy the space, which in turn is deeply related to abundance, on density related habitat selection or proportional-density model of no relationship between abundance and area occupied, supported by ideal-free distribution theory or the basin model, where positive abundance–area relationship, supported by density-dependent habitat selection theory, MacCall’s hypothesis. The relationship between occupancy and abundance is one of the most extensively studied patterns in macro-ecology [19,31,32]. However, this relationship has been rarely incorporated into surveillance or management [2,21,31]. Rindorf et al. [21] studied the biasness of several spatial indicators as a consequence of their relationship with abundance, using simulated data and analytical derivations and concluded that spatial indicators used for management should be unbiased for any level of abundance. Reuchlin-Hugenholtz *et al*. [2] found that changes in spatial indicators preceded rapid declines in fish biomass, thus these can be used as spatially based reference points in fisheries management whereas Adams et al. [33] found an effect of fishing pressure on the spatial distribution of marine fishes. Thus, there is a potential to develop spatial indicators based on the relationship between abundance and species spatial distribution.

With this in mind, the aims of the current work are (1) to select and present a shortlist of spatial indicators commonly used in marine studies, along with a bibliographic revision of the previous works that used those indicators (2) to analyze the relationship between the indicators, using empirical data and propose an unifying classification scheme for spatial indicators (categories) and (3) to integrate the information given by the categories of spatial indicators to inform on species ecology that can be used in management.

## Materials and methods

**Fig 1:**
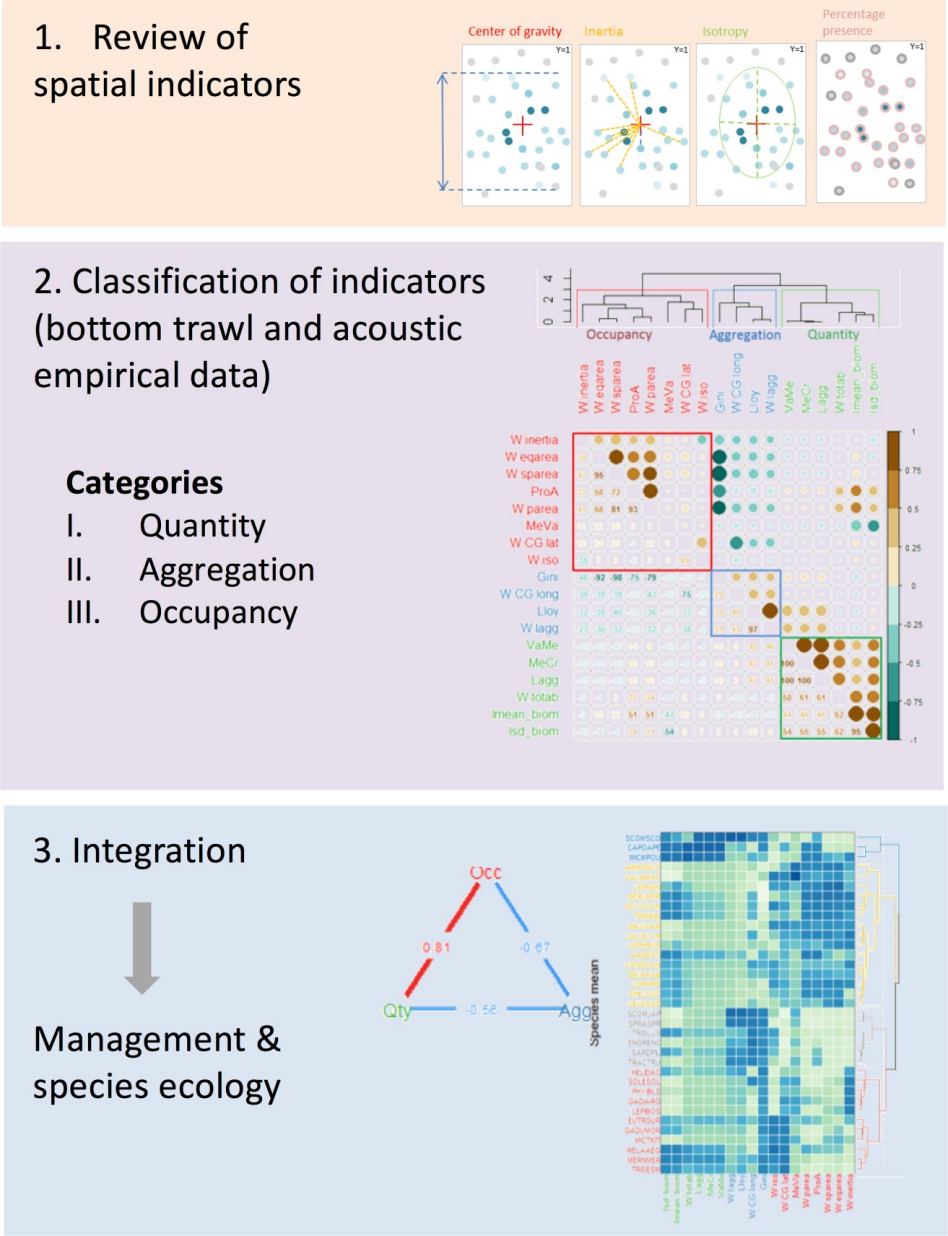
Graphical abstract. Conceptual diagram describing the three-steps approach of the present work (graphical abstract).

A general description of the work can be found in Fig 1.

### Data used

**Fig 2:**
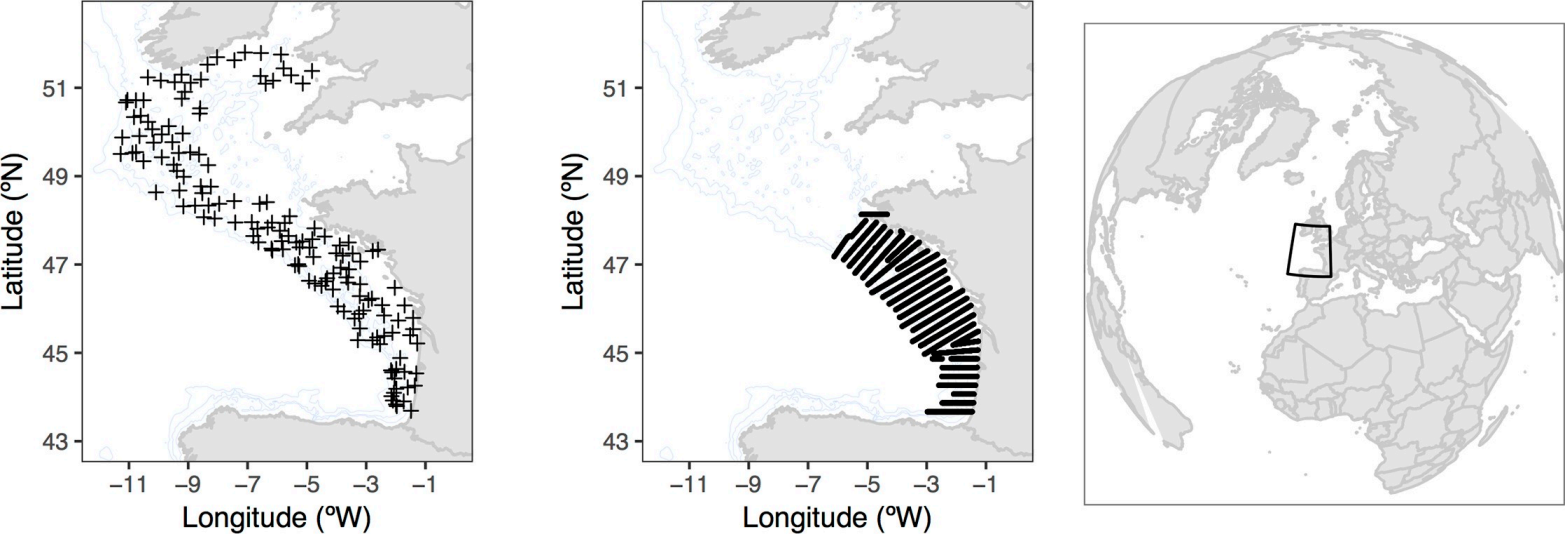
Location of the sampling stations. Location and sampling design of the empirical data sets used in the current work, bottom trawl survey (EVHOE 2015, left panel), small pelagic surveys using acoustic techniques (PELGAS 2015; middle panel) and worldwide reference of the areas (right panel).

The data was obtained from scientific groundfish bottom trawl surveys (demersal, FR-EVHOE) and pelagic acoustic surveys (PELGAS). EVHOE is a French international survey carried out annually during autumn in the Northeastern Atlantic to evaluate the demersal fishing resources [34]. It ranges from the Bay of Biscay up to the Celtic seas. The sampling is randomly stratified over 7 bathymetry intervals (0–30, 31–80, 81–120, 121–160, 161–200, 201–400 and 401–600 m) and is composed of 119 to 153 sampling stations per year (Fig 2, right panel). The biomass of the 29 fish species occurring more than 10 times per survey during the 19 sampled years (1997–2015) and excluding the main pelagic species was used: *Argentina* sp. (ARGENTI), *Arnoglossus* sp. (ARNOGLO), *Callionymus lyra* (CALMLYR), *Callionymus maculatus* (CALMMAC), *Capros aper* (CAPOAPE), *Chelidonichthys cuculus* (CHELCUC), *Conger conger* (CONGCON), *Eutrigla gurnardus* (EUTRGUR), *Gadiculus argenteus* (GADIARG), *Gadus morhua* (GADUMOR), *Helicolenus dactylopterus* (HELIDAC), *Lepidorhombus boscii* (LEPIBOS), *Lepidorhombus whiffiagonis* (LEPIWHI), *Leucoraja naevus* (LEUCNAE), *Lophius budegassa* (LOPHBUD), *Lophius piscatorius* (LOPHPIS), *Melanogrammus aeglefinus* (MELAAEG), *Merluccius merluccius* (MERLMER), *Merlangius merlangus* (MERNMER), *Microchirus variegatus* (MICUVAR), *Micromesistius poutassou* (MICPOU), *Microstomus kitt* (MICKIT), *Phycis blennoides* (PHYIBLE), *Scyliorhinus canicula* (SCYOCAN), *Solea solea* (SOLESOL), *Trisopterus esmarkii* (TRISESM), *Trisopterus luscus* (TRISLUS), *Trisopterus minutus* (TRISMIN) and *Zeus faber* (ZEUSFAB). Only most frequent species were considered, as species that are poorly caught by the survey gear will occur infrequently in survey catches and are therefore likely to create noisy indicator series [26].

The PELGAS acoustic surveys are carried out annually in spring, during the last 15 years (from 2000 to 2016); [35,36]. They consist on over 1345 to 1997 locations (Elementary Distance Sampling Unit (EDSU)), obtained along 29 acoustic radials perpendicular to the coast, used to evaluate small pelagic fish resources in the Bay of Biscay (Fig 2, middle panel). The six most abundant small pelagic fish species were used to study the indicators: *Engraulis encrasicolus* (ENGRENC), *Sardina pilchardus* (SARDPIL), *Scomber japonicas* (SCOMJAP), *Scomber scombrus* (SCOMSCO), *Sprattus sprattus* (SPRASPR) and *Trachurus trachurus* (TRACTRU).

### Spatial indicators

There are many ways of referring to the spatial organization of animals such as patch or patchiness, crowding, clump, pattern of variability, pattern intensity, spatial heterogeneity, spatial distribution, spatial structure, contagious behavior, convergence or aggregation and spatial distribution metrics [2]. Nevertheless, all these can be represented and quantified through spatial indicators. In the current work 18 spatial indicators commonly used in previous studies have been selected. The selection of indicators was based on their presence in the literature. We present them according to their sensitivity or not to the location of the samples in space. The first set of indices is spatially-independent and is divided into two subsets depending on whether the index is based on density measures or on count data. The second set is composed of spatially explicit indices, for which the geographical location of the sample matters. Table 1 provides selected references on the different uses of the indicators listed in this work. Also, a comprehensive table with an exhaustive listing of previous works using spatial indicators and respective synthesis can be found in Supplementary material (S1 File). This literature review was done to provide a broad overview on the previous applications of the spatial indicators, along with an identification of the potential pitfalls and future directions. The works considered in the review were selected using several internet search engines (google scholar, web of science, etc.) and citations on other articles. The indicators selected for this work do not intended to be exhaustive, but representative of those used in the literature.

**Table 1:** Summary of the indicators used in the current work, organized by group. Underlined indices are the ones retained for other analysis.

Indicator	Description	Scale	Code	Ref.
**1. Occupancy indicators**		**[0–1] (%)**		
Percentage of presence	% samples > 0 (independently of abundance)		ProA	proportion of empty samples (Rindorf *et al*. 2012) Saraux *et al*. (2014),
Positive area	The sum of the areas of influence of each sample (estimated using Voronoï) with positive densities (in nmi^2^).	0-total area	parea	Woillez *et al*. (2007) Woillez *et al*. (2009)
Equivalent area	The area that would be covered by the population if all individuals had the same density, equal to the mean density per individual [0-PosA](nmi^2^)	0-+ area	eqarea	Woillez *et al*. (2007) Woillez *et al*. (2009) Bez and Rivoirard [10]
Spreading area	Index related to the Gini index, but which has the advantage of having no contribution from zero values of density (nmi^2^).		sparea	Woillez *et al*. [11]
Inertia*	Describes the dispersion of the population around its center of gravity (nmi^2^)		Inertia	Bez and Rivoirard [10] Woillez *et al*. [11,12]
				
**2. Quantity dependent indicators**			
Coefficient of dispersion (σ^2^/mean ratio)	This index gives indications on over or under dispersion compared to a Poisson distribution.		VaMe	Bez and Rivoirard [10]; Szmyt [54]
Index of dispersion (contagion)	Used to measure the distributional pattern within the range (MSFD)		MeVa	Greenstreet *et al*. [14]
Level of aggregation	Mean density per individual, used to describe the level of aggregation.		Lagg	Bez and Rivoirard [10]
Mean crowding	Alternative indice to be used only with count data, which unlike Lloyd's index, it is not affected by zero counts (domain free)		MeCr	Bez [9];
Level of aggregation			Lagg	Bez [9];
Centre of Gravity*	Mean geographic location of the population (lat/long coordinates).		CG	Bez and Rivoirard [10] Woillez *et al*. [11,12]
				
**3. Aggregation indicators**				
Lloyds index of patchiness	Quantify the degree of patchiness.		Lloy	Rindorf *et al*. [21] Bez [9]
Gini (Lorenz curve)	Represents the difference between the observed distribution and a distribution where every sample contains the same individuals [0–1].		Gini	Woillez *et al*. [11] Rindorf *et al*. [21]
Index of aggregation	Describes the aggregation of the population.		Iagg	Bez and Rivoirard [10]
Isotropy*	Measures the elongation of the spatial distribution of the population.dispersion shape (symmetry) of the inertia around the center of gravity (i.e. round or ellipsoid), and it is the ratio between the two inertia axes. [0–1]		Iso	Woillez *et al*. [11,12]

* indicates the spatially explicit ones.

#### Spatially-independent indicators: Density based spatial indicators

The **percentage of presence** (ProA) is the ratio between the positive area and the surface of an area considered as the reference one. It is usually obtained by dividing the number of samples that contain at least one individual by the total number of samples, thus being a percentage (0–100% or 0–1)[2,16,21]. Crecco and Overholtz [32] considered instead the proportion of the survey area where catch rate was above a fixed level [21]. Persohn *et al*. [19] proposed a correction of this indicator to account for a stratified sampling design. Modica *et al*. [22] suggested to standardize the percentage of presence by the ratio between the survey area of each year over the largest one observed on the entire time series, to avoid a potential spurious effect of the existence of a different number of samples along the survey time-series. The percentage of presence can also be estimated using indicator kriging (geostatistics) applied to the data previously converted into presence/absence. This method provides estimates of the probability of presence, that are then translated into the percentage of presence by summing the surface of pixels exceeding a certain threshold of a probability (e.g. 50%)[13,37–39]. For the sake of simplicity, only the percentage of presence was considered in the posterior analyses.

The **positive area** (parea) is the area where a fish species occurs, i.e. the surface area of the geographical space occupied by a species, where its densities are strictly above zero [11,12,18,25,40,41]. It varies between zero and the total area. The positive area is highly sensitive to low density areas that have the same contribution as the high ones, like the percentage of presence. It can be estimated as the sum of the areas of influence around each positive sample [11,12]. As far as time series is concerned, if an area is added for a given year or if there are changes in the sampling design, the positive area can be highly impacted.

**Equivalent area** (eqarea) represents the surface that would be covered by a population with constant density equal to the mean density per individual [10,11,18]. This indicator is very sensitive to the highest density values [18,42].

**Spreading area** (sparea) measures how the densities of the positive area are statistically distributed [11,12,18,19,25,40,43](graphical representation in S2 File). It is related to the Gini index (explained below), but with the advantage of having no contribution of null densities. This indicator is much less sensitive to low values of density than is the strict positive area [18].

The **average biomass** (mean_biom; *μ*) and the **standard deviation** (sd_biom; *σ*) can also be considered as key indicators. Biomass is computed at each sample location and the average (or standard deviation) is calculated over all the samples.

The **level of aggregation** (Lagg) representing the mean density per individual has been proposed by Bez [9] and Bez and Rivoirard [10] to describe the level of aggregation of fish densities. The **index of aggregation** (Iagg) is then obtained by standardizing the level of aggregation by the total abundance (Bez and Rivoirard, 2001).

If the **equivalent area** (eqarea)(and so its inverse, the index of aggregation) remains practically constant, variations of abundance are compensated by variations of the level of aggregation (e.g. densities multiplied by a constant) [10,12,25,26,40]. On the other hand, if the level of aggregation is constant, variations of abundance are directly translated into variations of the equivalent area. Mixed situations can be thought of, where variations of abundance go along with variations of the level of abundance and of the equivalent area [10]. This indicator is very sensitive to the highest density values [18].

#### Spatially-independent indicators: Count based spatial indicators

**Lloyd’s index** of patchiness (Lloy) has been developed to quantify the degree of patchiness of count data at the scale of the sample support [9,10,21]. It is sensitive to zero abundance’s and is thus dependent on the domain over which it is computed (like all other indicators except CG, Lagg, Iagg and earea).

From Lorenz curves, two main indicators have been derived in previous works: Gini index and spreading area. Lorenz curves were initially developed in economics to estimate the concentration or richness/poverty (graphical representation in S2 File). When applied to fisheries, its abscissa represents the cumulative area arrayed by increasing biomass, and its ordinate, the corresponding proportion of the total fish biomass [44,45]. If fish abundance were equally distributed among the samples, the Lorenz curve would correspond to a 1:1 line. As the distribution of fish becomes increasingly uneven, i.e., more concentrated, the Lorenz curve bends downwards and to the right.

**Mean crowding** (MeCr) is an indicator proposed by Lloyd to be used with count data. Unlike Lloyd's index (shown above), it is not affected by zero counts (domain free)[9,46].

**Coefficient or index of dispersion** (VaMe), also called variance to mean ratio (σ^2^/μ), relative variance or Fano factor, is used to measure the spatial aggregation of individuals [14,46–52]. It consists on a normalized measure of the dispersion of a probability density function of count data [53]. Other related indicators used to measure dispersion in count data include Morisita’s index, Lloyd’s mean crowding (referred above), Green’s index and Taylor’s power law [47,51,54,55], not included for brevity but relevant to mention.

The **index of dispersion** (MeVa), also named as **mean to variance ratio** or index of contagion has been applied to measure “distributional pattern” within the occupied range for the MSFD [14].

**Gini index** (Gini) represents twice the area between the identity function and the Lorenz curve (also called concentration index or evenness of the spread). This index is commonly accepted as a measure of the concentration [2,11,12,21,44,56–60] (graphical representation in S2 File). It is bounded between 1 and 0, and the highest its value the most concentrated is the biomass in fewer samples.

The statistics VaMe, MeVa, Gini and Lloyd are all based on the statistical distribution of the sampled count data and are thus influenced by the zero counts observed outside the area of presence of fish (i.e. domain dependent), whereas mean density per individual (level of aggregation, Lagg), index of aggregation (Iagg) and equivalent area are domain free statistics, i.e. do not vary with the presence of external zeros in the sample data [10].

All previously mentioned indices are independent of how the values are actually spatially distributed, although are usually considered to be spatial indicators [10]. Indeed, if the location of the samples changes, it would not be reflected on the indicator’s results.

#### Spatially explicit indicators

**Centre of Gravity** (CG) (also referred as distribution centroid, center of mass, range center, range core, spatial core, and center of distribution) indicates the mean spatial location of the population (graphical representation in S2 File)[10,12]. When it is estimated with the coordinates only (mean latitude and longitude), it represents the center of the survey area. When weighted by abundances, the mean latitude and longitude become the coordinates of the center of the population [10]. The latter version was computed in the present work.

The **inertia** (Inertia) represents the spatial dispersion of the population around its center of gravity, i.e. the mean square distance between individual fish and the center of gravity [10,12], also called geographical spread index [22] (graphical representation in S2 File). Other works have estimated the variability of the bivariate distribution using ellipses and confidence intervals or kernels (see table S1 File).

**Isotropy**/anisotropy (isotropy) represents the dispersion shape (symmetry) of the inertia around the center of gravity (i.e. round or ellipsoid), and it is simply the ratio between the two inertia axes (graphical representation in S2 File).

In the case of irregular sampling as randomly stratified sampling design, typically the case of fishing surveys, some of the above indicators can optionally take into account the areas of influence around each sampling point (namely the center of gravity, inertia, isotropy, positive area, spreading area, index of aggregation and equivalent area)[12]. This area of influence around each sample can then be accounted for as a constant (for example 1 or the area of the grid square) or having a variable area of influence estimated around each sample. In this latest case previous works suggest that the areas of influence are estimated using Voronoï tessellation [12]. This step is always carried out prior to the calculation of the indicators. The results of six indicators estimated accounting for different areas of influence (constant *vs*. Voronoï tessellation) were compared using linear models. Additionally, as the areas of influence are most commonly estimated using discrete Voronoï tessellation, we also tested the two parameters required in their computation: the maximum distance allowed between data and borders (called *dmax*) and the level of discretization (called *nodes*). The effect of those two parameters has been evaluated by varying the two parameters from 0 to 500, and looking for a stabilization of the indicator values (i.e. plateau). Values at the start of the plateau was deemed the best values to use for the parameterization of the Voronoï function in our case. All those results are shown in detail on supplementary material (S3 File).

### Classification of indicators

To classify the indicators into categories using empirical data, the results given by the fifteen spatial indicators were first applied to 569 species raw biomass distributions (15/19 years, 2 surveys and 31 fish species). For these, when applicable, the indicators were weighted by the areas of influence estimated by Voronoï (as indicated previously). Thus, the matrix of indicators, together with summarizing measures of the species abundances (average biomass (log (mean_biom+1)), respective standard deviation (log (sd_biom+1)) and total biomass (log (totab+1)) were explored using a principal component analysis (PCA), a correlation matrix plot and a hierarchical clustering to determine the main groups of indicators, and the relationships among those.

The indicators were then averaged at species level (across years), and were represented using a heatmap with the respective clusters that arose from the correlations. A PCA (produced on scaled variables), allowed determining the groups of species showing similar patterns across the indicators.

### Integration of the indicators in ecology & management

Based on the results of the previous section, we selected one indicator per category, namely the percentage of presence, the Gini index and the average biomass. For each species, Spearman rank correlation (ρ) was calculated among the time series of the three indicators to diagnose the existence of a significant monotic relationship. Significant correlations (p-value ≤ 0.05) were then represented using a triangular network with three nodes (or vertices) where the edges (or links) were proportional to the strength of the rank correlation (ρ) and colored accordingly. The rank correlation matrix was analyzed with hierarchical clustering and k-mean approaches to define groups of species with similar spatial behavior.

All analyses and plots were performed using the statistical programming language R. Spatial indicators were estimated self-coded or using existing packages RGeostats [61] and ineq (for Gini index) [62]. Figures were produced using the packages ggplot2 [63], superheat [64], factoextra [65] and corrplot [66].

## Results

### Literature review

The brief literature review resulted in 55 studies using spatial indicators in marine ecology and published in the last 20 years. The studies are listed and summarized accordingly (table with respective references and classification in S1 File). Spatial indicators have been applied essentially to study the spatio-temporal species dynamics (72% of the studies; spatio-temporal dynamics and model based), but also on model validation (comparing IBM and similar models with survey data, 7 studies) and spatial overlap of species distribution (4 studies).

The indicators proposed by Woillez et al. [12], also considered in the current work, dominate the literature (47 out of 55 studies). The selection of spatial indicators varied widely among studies, although the center of gravity with the respective inertia was one of the most frequently used (46 studies). Furthermore, most studies used few indicators and only 8 studies integrated 7 or more spatial indicators to describe the spatial distributions of populations.

Forty-seven studies used at least one of the 6 indicators that require the areas of influence, as recommended in Bez and Rivoirard [10] and Woillez et al. [12]. Out of these, in 24 of those studies the weighting by the areas of influence was not mentioned, whereas in 6 studies, it was mentioned but no clear details on how it was done were given. Four studies stated that the areas of influence were estimated using Voronoï (or Dirichlet tessellation) and only one, provided the details used in their estimation.

Our results indicate that the required parameters used to estimate the areas of influence around each sample can have a large impact on the results given by the indicators when they are not crude (see supplement for details). Still, whether the indicators were weighted by sample’s specific areas of influence or a constant, the given results were highly correlated for the two datasets considered. For the center of gravity, inertia and isotropy the relationship was similar for both surveys whereas for the positive area, equality area, spreading area and index of aggregation, the relationship was strong, but differed between surveys.

### Classification of indicators

**Fig 3:**
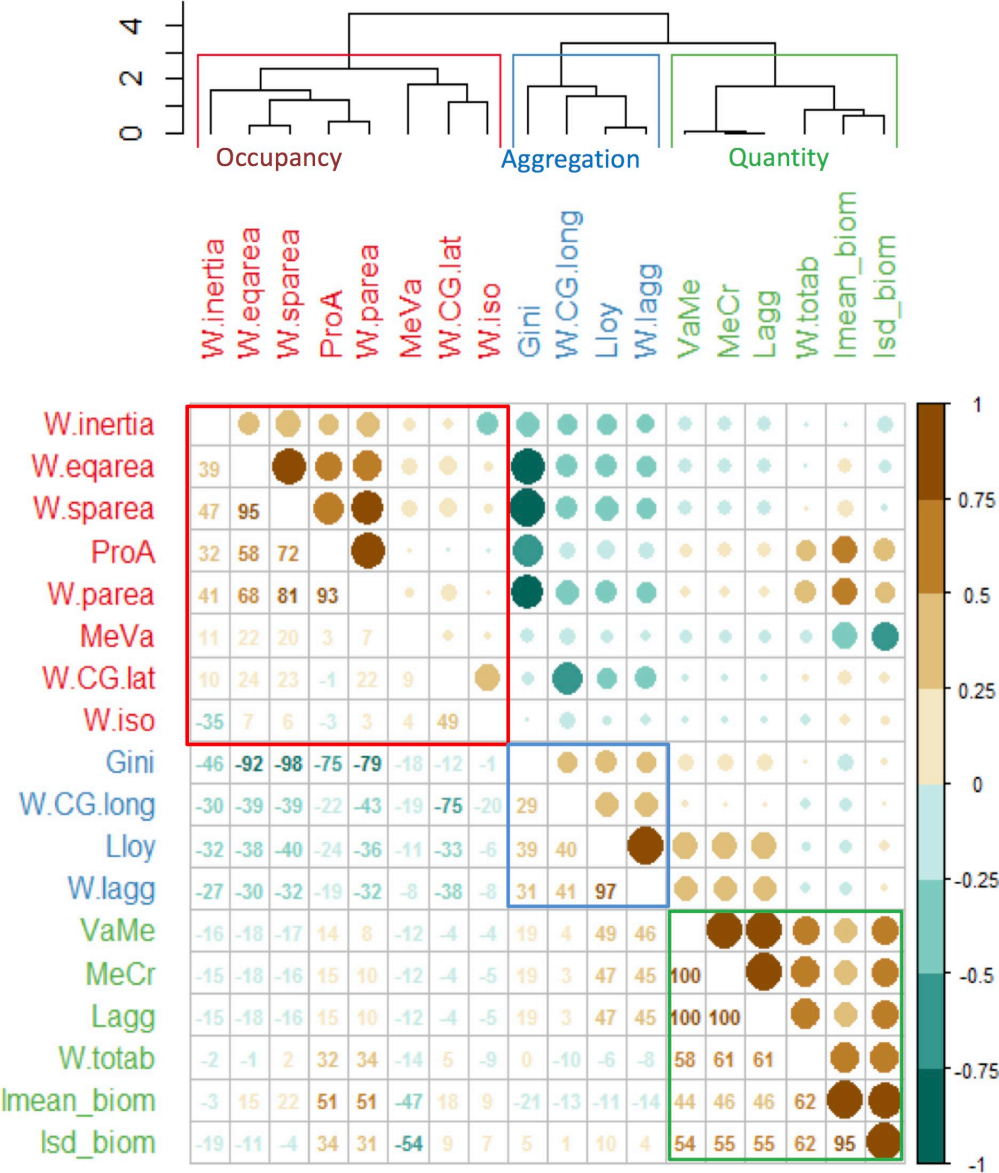
Relationships among spatial indicators. Correlation matrix plot of the indicators estimated for each annual species distribution, and respective cluster groups (on top) that represent the three categories (colored accordingly).

Three groups of highly correlated indicators were evidenced in the cluster analysis, the correlation matrix plot and the PCA (S4 File and Fig 3) using the 18 indicators applied to the full time-series of the 35 species. The resulting groups of indicators highlighted different aspects of the species spatial distributions.

The first group of indicators, included species average biomass and standard deviation and represented a category of quantity-dependent indicators (S4 File and Fig 3 group ‘Quantity’). It comprised the variance to mean ratio (VaMe), mean crowding (MeCr), level of aggregation (Lagg), total biomass (totab), the mean species biomass and respective standard deviation (lmean_biom and lsd_biom) (average intra-group correlation of 66.6%).

The second group of indicators represented the aggregation indicators category that is, whether the species distribution is more concentrated or more spread in space (S4 File and Fig 3, group ‘Aggregation’). This group included Lloyd’s, Gini’s and the index of aggregation (Iagg), all highly positively correlated (average intra-group correlation of 46.2%). Further, x coordinate corresponding to the longitude of the center of gravity was also within this group (CG.long).

The third group of indicators represented occupancy indicators category, and reflected whether the species were found in most of the samples, thus spread over the entire area or just in part of it (S4 File and Fig 3, group ‘Occupancy’). This category included inertia (W.inertia), positive area (W.parea) and percentage of presence (ProA), spreading area (W.sparea) and equivalent area (W.eqarea) (average intra-group correlation of 63%). Within this group, inertia was the only spatially explicit indicator. Further, this group also included a sub-group with the mean to variance ratio (MeVa), the y coordinate corresponding to the latitude of the center of gravity (W.CG.lat) and isotropy (W.iso), the latter contributed poorly to the ordination. This latter sub-group however, showed reduced correlation levels with all indicators overall.

The first PCA axes separated the species with higher values for the occupancy indicators from the species showing higher value for the aggregation indicators, such as those sampled during the pelagic survey (S4 File, left and middle panel and Fig 3). The second axes showed mainly the influence of quantity based indicators (S4 File, left panel and Fig 3). Analysis of the inter-annual variability in species distributions suggested that the species are spatially stable in their distribution and that the diversity of distributional patterns are likely species specific (PCA in S4 File). Thus, the indicators were averaged over the species (across years) and the analysis redone.

**Fig 4:**
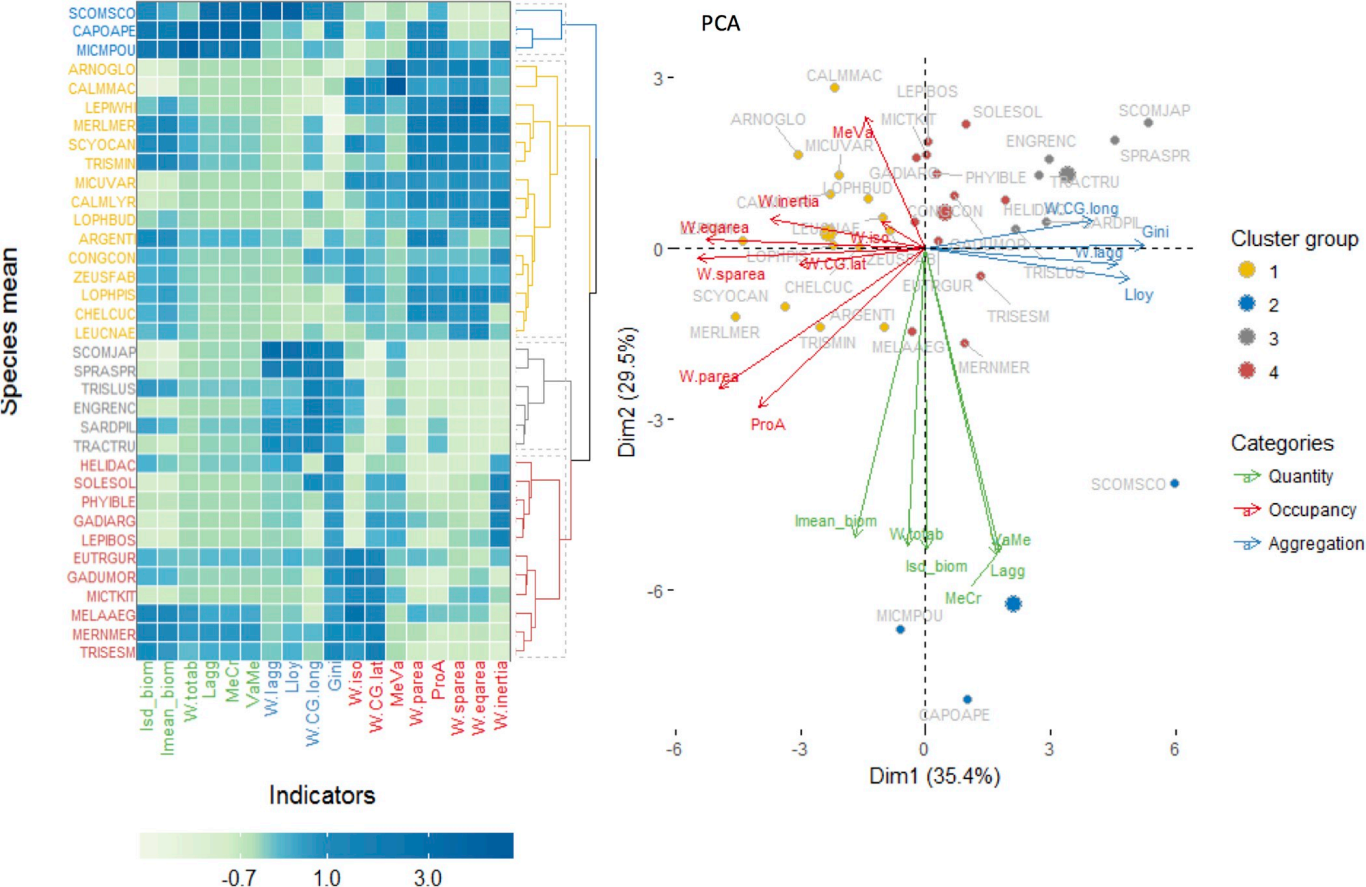
Species groups according to the indicators. The results of each indicator for all years, were averaged by species and scaled. Left panel represents the heatmap with respective cluster (groups defined by kmeans) and right panel, shows the PCA results. Indicators and species names/symbols are colored according to the categories or cluster groups, respectively.

The results of the averaged indicators by species also evidenced the same three groups of indicators as it was found in the full matrix (i.e. including annual variability), but it also identified four main groups of species, as shown in the heatmap (Fig 4). The first group was represented by species with high results for quantity indicators (*Scomber scombrus*, *Capros aper* and *Micromesistius poutassou*; blue group in Fig 4). The second group was composed of species with high values for occurrence indicators and lower aggregation indicators (*Arnoglossus* sp., *Callionymus lyra*, *Callionymus maculatus*, *Lepidorhombus whiffiagonis*, *Merluccius merluccius*, *Scyliorhinus canicula*, *Trisopterus minutus*, *Micromesistius poutassou Lophius budegassa*, *Argentina* sp., *Conger conger*, *Zeus faber*, *Lophius piscatorius*, *Chelidonichthys cuculus* and *Leucoraja naevus*; yellow group in Fig 4). The third group was mostly associated with species captured in the pelagic survey and included species that showed high aggregation indicators and lower occurrence (*Engraulis encrasicolus*, *Sardina pilchardus*, *Scomber japonicas*, *Sprattus sprattus* and *Trachurus trachurus*; grey group in Fig 4). The fourth group was composed of species captured in the bottom trawl survey, showing relatively higher values for the aggregation indicators (red group in Fig 4), in particular for Gini index (*Helicolenus dactylopterus*, *Solea solea*, *Phycis blennoides*, *Gadiculus argenteus*, *Lepidorhombus boscii*, *Eutrigla gurnardus*, *Gadus morhua*, *Microstomus kitt*, *Melanogrammus aeglefinus*, *Merlangius merlangus* and *Trisopterus esmarkii*).

### Integration of the indicators in ecology & management

Based on the results from the previous analysis, one indicator per category was selected for the posterior analysis. The choice rested on the simplicity of calculation of the indicator, its straightforward interpretation, and its general use and comparability among species (two of them are percentages, i.e. bounded). Thus, for the occupancy category, percentage of presence was selected, for the aggregation indicators, the Gini index was considered and to represent quantity derived indicators, the average biomass was used. Those measures were used to characterize the quantity-occupancy-aggregation relationships, and thus how each species occupy the space.

**Fig 5:**
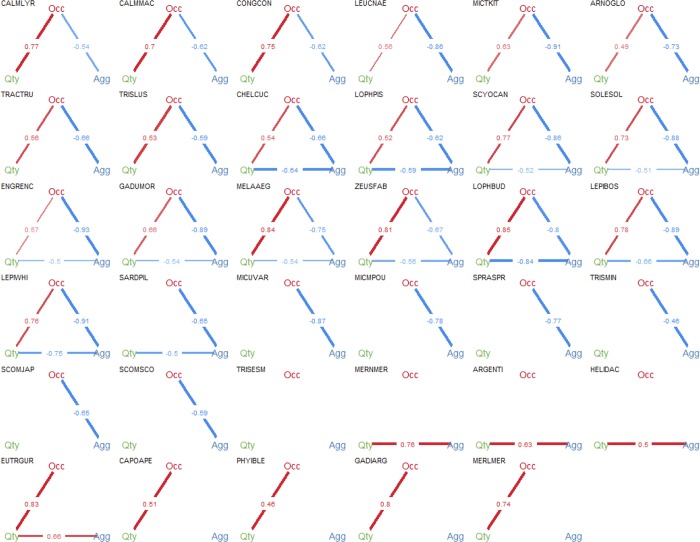
Relationships between quantity-occupancy-aggregation for all species under study. Spearman correlation between the three aspects of species spatial behavior (years pooled): Aggregation (measured by Gini index), Occupancy (measured by the percentage of presence) and Quantity (measured by the mean log biomass). The width of the edge line represents the strength of the relationship, its color the direction (positive in blue and negative in red) and the value in the middle, is the correlation coefficient. Non-significant correlations were omitted. Codes for the species can be found in the text.

**Fig 6:**
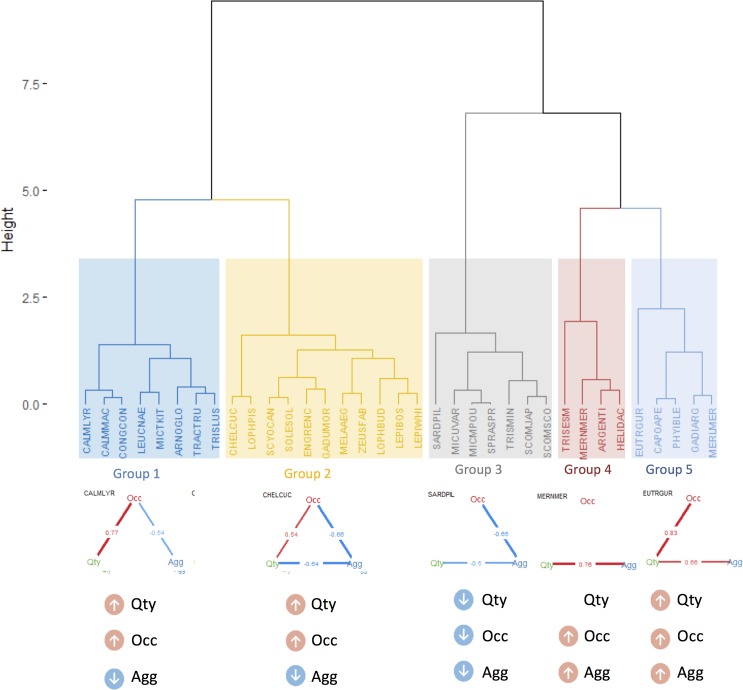
Groups of species with similar quantity-occupancy-aggregation relationships. See further details in the text.

For the 35 species considered, only one (*Trisopterus minutus*) did not show any significant relationship between quantity-occupancy-aggregation, as measured by Spearman correlation (Fig 5 and Fig 6). All the remaining species were grouped by the dominant relationships among those three axes.

The first group of species showed a positive significant relationship between quantity and occupancy and a negative relationship between occupancy and aggregation (Fig 5 and group 1 Fig 6). Therefore, for those species, in years of greater quantity, there was an expansion of the area occupied, which in turn corresponded to a decrease in the aggregation within the sampled areas. A second group showed a similar pattern to group 1, with, however, an additional significant negative relationship between quantity and aggregation (Fig 5 and group 2 Fig 6). This additional link suggested that in years of greater abundance, the species are more clustered. A third group showed only one significant relationship between occupancy and aggregation, but no significant relationship between quantity and the other two variables. Therefore, in these group, when occupancy increases aggregation decreases, independently of the quantity which is not significantly related with any of those (Fig 5 and group 3 Fig 6). A fourth group showed only one significant positive relationship between quantity and aggregation, but no significant relationships between occupancy and the other two variables. Therefore, in these group, when quantity increases, aggregation also increases independently of occupancy (Fig 5 and group 4 Fig 6). A fifth group showed a significant positive relationship between quantity and occupancy and another between quantity and aggregation, but no significant relationships between aggregation and occupancy Therefore, when quantity increases, occupancy also increases (Fig 5 and group 5 Fig 6).

## Discussion

Spatial indicators are used to quantify the ecological states of species, the impact of climate change and other anthropogenic aspects that are likely to be reflected in their distribution, but also to differentiate the anthropogenic-induced distribution from their natural variability [10,11,14,21,26,30,41,67]. They are considered as potentially key tools for decisions makers, although hardly any previous work have focused on their applications in management [14,30,68,69]. Our study underlined that the results given by 18 different spatial indicators were highly redundant, representing essentially three aspects of species spatial distribution: quantity, aggregation and occupancy. Those three components represented species spatial behavior, and thus can be used to classify the species in each area and the relationships between the three aspects can be summarized in a triangular diagram. This representation defines the way a species occupy the space in terms of occupancy and aggregation and its relationship with quantity. This approach can be further used in management and ecology in future works.

To the author’s best knowledge, the only previous quantitative literature review on spatial indicators has been recently done by Yalcin and Leroux [30], focusing on indicators used to quantify species ‘range’. Additionally, Adams et al. [33] made a short synthesis of previous works that used the center of gravity. Here the literature review permitted to assemble the conclusions of those two abovementioned studies, and extended them to more spatial indicators (S1 File). Previous studies on spatial indicators are essentially focused on species spatial-temporal dynamics, namely with its relationship with temperature/fishing effort or between species life-stages. From our review it becomes clear that spatial indicators are commonly used in marine biology studies, in a broad diversity of areas.

For irregular sampling designs it has been recommended that the indicators are weighted by the areas of influence around each sample [12]. However, the majority of the previous works reviewed (S1 File) did not mentioned if the calculations incorporated an estimation of the areas of influence around each sample, whereas when it is mention, the parameters considered were not specified except in Doray et al. [28] (S1 File). This underlines at best a failure in the methodological description or at worst a misunderstanding of the functions used to estimate the indicators. In the current work we showed that the parameters used to estimate the areas of influence around each sample can have a large impact on the results given by the indicators when they are not appropriate. This leads to two recommendations: first, it is essential to carry out a simple preliminary study to identify the best values of the parameters. In the current work we are proposing a simple technique to parameterize the areas of influence estimated by Voronoï, which can be extended to any method applied. Second, the parameters specifications should be detailed in future works, otherwise the results will hardly be comparable.

Nevertheless, those particularities, it was concluded that whether the indicators were weighted by sample’s specific areas of influence or a constant, the given results were highly correlated for the two datasets considered. For the center of gravity, inertia and isotropy the relationship was similar for both surveys whereas for the positive area, equality area, spreading area and index of aggregation, the relationship was strong, but differed between surveys.

Ideally, spatial indicators should be independent of the sampling scheme. Still, this is not the case for estimators of the spatial range neither for the percentage of non-empty samples [22,67]. Most spatial indicators considered in the current study have been previously applied to fish data obtained by bottom trawl or by acoustic surveys, but not between these two types of surveys in an integrative perspective, as it is done in the current work (S1 File). Thus overall the relationships between the indicator’s results did not differed between the two different sampling designs considered (randomly stratified of the trawl survey and systematic transects of the acoustic survey).

### Classification of the indicators

When many correlated indicators are used for monitoring purposes, they may show conflicting signals that are not interpretable, or over emphasize the seriousness of the situation [24]. Barra *et al*. [25] found that small pelagic fish biomass was significantly related to a suite of, rather than single, spatial indicators, thereby extending the abundance-occupancy relationship to other aspects of species’ spatial behavior, which is also observed by Reuchlin-Hugenholtz *et al*. [2] and confirmed in the current work. However, we conclude that this can be due to the correlation between indicators and not to a particularity of the species studied. In the current work, it was observed using empirical data that most indicators were highly correlated with each other. Nevertheless, these could be disentangled into three main groups of indicators that define the species spatially: quantity, aggregation and occupancy. It is important to mention that a strong correlation between two indicators does not imply that they are measuring the same ecological aspect of a species but that the values of the species spatial distributions are similar. To illustrate this clearly, consider the width and height of an animal. These two variables are strongly correlated between each other and are both proxies of size, despite representing a different measure of shape.

An unified classification scheme of spatial indicators quantifying a diversity of species distribution, would improve the comprehension of its meaning, permit the development and implementation of surveillance indicators and can facilitate the communication with stakeholders [5,70,71]. Woillez *et al*. [12] considered eight classes of spatial indicators to characterize species spatial distribution: location (latitude and longitude of the center of gravity), dispersion (inertia and anisotropy), aggregation (spreading area), occupation (positive area) and correlation (microstructure). Rindorf *et al*. [21] however, referred to three classes only, to classify eight spatial indicators.: area occupied (proportion of empty samples and structurally empty samples), aggregation (Lloyds index and Lorenz curves), and area spread or range (average distance to the center of gravity and area of the contour ellipse). Our results also defined three main classes of indicators, that have an ecological meaning: biomass, spatial aggregation and occupancy.

Furthermore, in the current work we underlined that the spatial behavior is highly species specific, as its inter-annual variability is smaller than the variability shown between species. Species spatial behavior primarily followed variations between occupancy and aggregation (reflected in the first PCA axes), and secondly reflected quantity (second PCA axes), as measured by the spatial indicators considered. In fact, species can be classified per their spatial behavior considering all indicators measured, and can be highly aggregated (e.g. pelagic species as the sardine) with lower ocupancy or occupy larger areas, but more spread, with lower aggregations (such as *Scyliorhinus canicula* or *Merluccius merluccius*), independently of its bathymetric range or area occupied within the sampled zones.

### Integration of indicators in management and ecology

In many previous works, spatial indicators have been used to disentangle species spatial behaviour, to test abundance–occupancy relationships (AORs) (S1 File). Several ecological-consistent theories have been developed around these concepts, namely the ideal free distribution theory (IFD) that predicts that biological populations contract into areas of highest habitat suitability as their abundance decreases, advocating therefore the existence of a relationship between abundance and occupancy. Similarly, MacCall’s basin model which postulates that geographic range of marine fish will co-vary with population density as a function of habitat selection [2,19,31,49,72–76]. A decrease in the area occupied at low stock abundance has been reported for many marine species, translated in a decrease in catchability [13,58]. Density-aggregation relationships have been reported for lakes invertebrates, across spatial scales and seasons [77]. Rindorf *et al*. (2012) confirmed that several spatial indicators were biased for different levels of abundance. Adams et al [33] used the positive area as a measure of occupancy to study the density-occupancy relationships of nine fish stocks, but also argues about this indicator reliability, as a zero may represent a low probability of capture. Thus, several previous works have focused on either density-occupancy or density-aggregation relationships. Nevertheless, no matter what the chosen indicators within each category, our results indicate that those two relationships should be interpreted together, and thus the species behavior characterized by a minimum of three axes: density, occupancy and aggregation. Therefore, it is suggested that the three axes of species behavior should be integrated in ecological monitoring. Similar conclusions have been reported by Hui et al [78] using stream macroinvertebrates and ants, that stated the use of the three distinct but related concepts of population structure (i.e. occupancy, abundance and aggregation) in conservation biology and produces a theoretical model to apply it. Zwanenburg et al [79] reached similar conclusions studying seven fish species, but named the three components differently: concentration, prevalence and local density. These authors also consider that the three measures should not be interpreted individually, but together, over time in each area of interest. Further work is required to develop a method to integrate quantity, occupancy and aggregation in marine species management, and to better define which indicators should be used in each case. Here, the integration was done using the simplest indicators, independently of their mathematical and theoretical criteria as it most likely should. Further work on the pitfalls and sensitivity of each indicator could also point to alternative criteria for the selection of indicators. Nevertheless, we would expect that the results would be similar in face of the strong correlations found within each category.

No single indicator is sufficient to summarize the spatial behavior of an animal [22] and scientific judgments remain essential for the selection and interpretation of survey-based indicators and assessments, depending on the biology of the stocks, the ecosystem, and the history of fishing [26]. Nevertheless, objective and optimized decisions supported by indicators must be made to preserve the environment. Thus, it is essential to promote the existence of well-studied, robust and accurate indicators of species distributions. The need for rigorous selection procedure to identify the minimum number of indicators necessary to support management is essential [7]. Indicators can then be used to pinpoint changes in the species and ecosystems that are then further studied to identify the nature of those shifts, so that effective management measures are developed and implemented.

## Supporting information

S1 File Review of previous works using the spatial indicators. Works are organized by subject, with a brief quantitative description on the indicators used, the inclusion of the areas of influence in its calculations (if it is mentioned and whether the details for calculation were provided), the species, temporal interval considered, type of sampling and area studied. Spatial indicators: CG: center of gravity; I: inertia; PA: positive area; EA: equivalent area; SA: spreading area; IC: index of collocation (either global or local); SP: spatial patches; G: Gini; MS: microstucture index; IA: index of aggregation; OC: occupied area; Sampling: bts = bottom trawl surveys; as = acoustic surveys; Note that Voronoi was considere to be equivalent to Dirichlet tessellation.
(DOCX)

S2 File Illustration of spatial indicators. Illustration of some spatial indicators mentioned in the text: Gini index, Lorenz curve and spreading area (upper panel), center of gravity, inertia and isotropy (lower panel).
(DOCX)

S3 File Options on the estimation of the areas of influence. (DOCX)

S4 File PCA with complete matrix (species and years). (DOCX)
