# Supplementary material for: Integrating spatial indicators in the surveillance of exploited marine ecosystems
Source: PLoS One. 2018 Nov 21;13(11):e0207538. doi: 10.1371/journal.pone.0207538 (PMC6248972; doi:10.1371/journal.pone.0207538)
Supplement: S1 File — Works are organized by subject, with a brief quantitative description on the indicators used, the inclusion of the areas of influence in its calculations (if it is mentioned and whether the details for calculation were provided), the species, temporal interval considered, type of sampling and area studied. Spatial indicators: CG: center of gravity; I: inertia; PA: positive area; EA: equivalent area; SA: spreading area; IC: index of collocation (either global or local); SP: spatial patches; G: Gini; MS: microstucture index; IA: index of aggregation; OC: occupied area; Sampling: bts = bottom trawl surveys; as = acoustic surveys; Note that Voronoi was considere to be equivalent to Dirichlet tessellation. (DOCX) [file pone.0207538.s001.docx]

### Supplement 1

- **Brief interpretation of the table:**

In terms of the species summarized by the spatial indicators, 49 works were focused on fish (31 with one species and 17 with more than two) and only four works focused on invertebrates (cephalopods and crustaceans). Spatial indicators have been used primarily to summarize longer time series, as only 4 works accounted for 1 to 2 years, whereas in 32 works, more than 10 years of data were integrated. In terms of the sampling device used, in 25 works the data was sampled during bottom trawl surveys, in 10 of them, the main data source was based on acoustic sampling and in five, plankton and eggs sampling was carried out. In terms of geographic origin, most works were located on the North-East Atlantic area, including North Sea, Bay of Biscay-North-West Ireland, Iberian coast, English Channel and in the North-West Atlantic, namely Georges Bank, Gulf of Maine, Gulf of St. Lawrence and Newfoundland (9 works). Six works focused on Mediterranean and other 6 on the North Pacific. Only one work integrated data from surveys taken in different parts of the world, but it used a model-based approach to estimate the indicators.

Besides changes in the species spatial-temporal distribution, these indicators have been used to address the effect of climate change, bottom temperature, other oceanographic indices and fishing effort. The spatial indicators have also been used to compare the prey and predator distributions, different life stages of a species (e.g. larval stages, juveniles, adults or different ages/cohorts) and co-existing species (e.g. sardine and anchovy). Often, spatial indicators have been used to explore species density-occupancy relationships, test for MacCall’s hypothesis or for the Ideal Free Distribution Theory. Another common previous application of the spatial indicators has been in model validation (e.g. individual model, ecosystem models, fisheries models), by comparing the spatial distribution of the modelled results with survey data (reality).

S1 Table : Table of previous works using the spatial indicators (literature review), by subject, with a brief quantittaive description on the indictators used, the inclusion of the areas of influnce in its calculations (if is is mentioned and wether the details for calculation were provided), the species, temporal interval considered, type of sampling and area studied. Spatial indicators: CG: center of gravity; I: inertia; PA: positive area; EA: equivalent area; SA: spreading area; IC: index of collocation (either global or local); SP: spatial patches; G: Gini; MS: microstucture index; IA: index of aggregation; OC: occupied area; Sampling: bts=bottom trawl surveys; as=acoustic surveys; Note that Voronoi was considere to be equivalent to Dirichlet tessellation.

| Reference | Subject | Indicators used | Areas of influence | Species (groups) | Period | Sampling | Area |
| --- | --- | --- | --- | --- | --- | --- | --- |
| Petitgas (2009) | methods description | CG, I, i, PA, SP, EA, MS | not mentioned | *Gadus morhue* (different ages) | 1985-2005 | bts (DA-TRAS-ICES) | North Sea |
| Woillez, Rivoirard, and Petitgas (2009) | methods description | CG, I, i, IC, PA, SP, EA, MS, SP | Voronoi, but not detailed |  |  |  |  |
| Adams et al. (2018) | spatio-temporal dynamics | CG, I, PA | Voronoi, but not detailed | 9 fish sp | 1963-2016 | bts | Northwest Atlantic |
| Adams (2017) | spatio-temporal dynamics | CG, I, PA | Voronoi, but not detailed | *Peprilus triacanthus (fish)* | 1982-2013 | bts | Northeast US |
| Alvarez et al. (2004) | spatio-temporal dynamics | CG, distribution ellipses | not mentioned | *Merluccius merluccius* (fish eggs and larvae) | 1995-1998 | Bongo (ICES plankton surveys) | Bay of Biscay to the north-west of Ireland. |
| Alvarez et al. (2001) | spatio-temporal dynamics | CG, distribution ellipses | not mentioned | *Merluccius merluccius* (fish eggs and larvae) | 1983-1995 | Bongo (ICES plankton surveys) | Bay of Biscay |
| Atkinson et al. (1997) | spatio-temporal dynamics | statistical ellipses, area occupied | not mentioned | *Gadus morhue* | 1981-1993 | bts | Newfoundland |
| Barra et al. (2015) | spatio-temporal dynamics | CG, NP, I, i, PA, AS, EA, IC | areas of influence from gridding | *Sardina pilchardus* and E*ngraulis encrasicolus* | 2002-2010/2004-2006 | as (MEDIAS) | Strait of Sicily and North Aegean Sea, |
| Baudron and Fernandes (2015) | spatio-temporal dynamics | CG | not mentioned | *Merluccius merluccius* | 1978-2011 | bts | North Sea, West of Scotland, Celtic Sea, Porcupine bank and Bay of Biscay |
| Bez and Rivoirard (2000) | spatio-temporal dynamics | CG, I | not mentioned | *Scomber scombrus* (eggs/larvae) | 1989 | as (ICES) | North-east Atlantic (Bay of Biscay, Celtic Sea, and west of Ireland). |
| Bitetto et al. (2012) | spatio-temporal dynamics | CG, IC, I, PA SP | not mentioned | *Aristaeomorpha foliacea* (crustacea) | 1994-2010 | bts (MEDITS) | Central-southern Tyrrhenian Sea (GSA) |
| Blanchard et al. (2005) | spatio-temporal dynamics | area occupied (#ices rectangles) with >95% of population | not relevant | *Gadus morhue* (juvenile) | 1977-2012 | bts | North Sea |
| Bonanno et al. (2017) | spatio-temporal dynamics | PA, SA, EA, CG, I, i, SP | not mentioned | *Engraulis encrasicolus, Sardina pilchardus, Sardinella aurita, Trachurus trachurus* and *Boops boops* | 2006-2015 | as | Central Mediterranean Sea (Sicily) |
| Bourdaud et al. (2017) | spatio-temporal dynamics | IC | not relevant | several sp | 1998-2014 | bts (CGFS)/onboard sampling (OBSMER) | English chanel |
| Boyra et al. (2013) | spatio-temporal dynamics | CG | not mentioned | *Engraulis encrasicolus* (juvenile) | 2003-2010 | as (JUVENA) | Bay of Biscay |
| Bruge et al. (2016) | spatio-temporal dynamics | CG, P5, P95 | not mentioned | *Scomber scombrus* (Pres/Abs data and egg production ) | 1992–2013 | eggs survey | EU North Atlantic coast |
| De Robertis and Cokelet (2012) | spatio-temporal dynamics | CG | not mentioned | *Theragra chalcogramma*/zooplancton | 2007-2008 | as/commertial vessels | Bering Sea |
| Engelhard et al. (2011) | spatio-temporal dynamics | CG, Weighted standard deviations and standard errors of the weighted mean latitudes were calculated | not mentioned (gridded) | *Solea solea; Pleuronectes platessa* | 1913-2007 | catch and effort data for British North Sea trawlers | North Sea |
| Faraj and Bez (2007) | spatio-temporal dynamics | CG, I, i, IA | mentioned but no detail given | *Octopus vulgaris* | 1998-2004 | bts | Atlantic coast of Marrocco |
| Gastauer, et al. (2016) | spatio-temporal dynamics | CG, I, i, PA, EA, G, IC | mentioned but no detail given | *Micromesistius poutassou* | 2006–2014 | as (IBWSS) | West of British Isles and Ireland |
| Honkalehto et al. (2011) | spatio-temporal dynamics | CG, I, IC | not mentioned | *Theragra chalcogramma* | 2006-2009 | Acoustic-trawl, bottom trawl and acoustic data collected from commercial fishing vessels | Eastern Bering Sea |
| Hughes et al. (2014) | spatio-temporal dynamics | CG, I, i | not mentioned | *Scomber scombrus* (eggs) | 1977-2010 | eggs survey (NEA- WSC; ﻿plankton tows) | North-west Atlantic |
| Modica et al. (2016) | spatio-temporal dynamics | %presence, CG, I | not mentioned | *Helicolenus dactylopterus*, *Merluccius merluccius* and *Lophius budegassa* | 1983-2010 | bts (DEMERSALES) | southern Bay of Biscay |
| Morfin et al. (2012) | spatio-temporal dynamics | CG, I, presence area | not mentioned | 12 sp | 1994-2010 | bts (MEDITS) | Gulf of Lions |
| Murawski, Finn and Finn (1988) | spatio-temporal dynamics | CG,Green's index of spatial dispersion; | not mentioned | 7 fish sp/different ages | 1963-1981 | bts (NEFC-NMFS) | Georges Bank |
| Myers et al. (1995) | spatio-temporal dynamics | G | by strata | *Hippoglossoides platessoides*, *Reinhardtius hipppoglossoidcs*, *Glyptocephalus cynoglossus* and *Lirnanda ferruginea* | 1971-1994 | bts | Newfoundland |
| Paulino et al. (2017) | spatio-temporal dynamics | CG, I | not mentioned (gridded) | *Dosidicus gigas* (cephalopod) | 2004-2015 | satelite luminosity | Peru |
| Perry et al. (2014) | spatio-temporal dynamics | mean latitude (center of distribution) | not mentioned | 36 fish sp | 1977-2001 | bts | North Sea |
| Persohn, Lorance and Trenkel (2009) | spatio-temporal dynamics | area occupied (adapted spreading area) and occurrence, ﻿cumulative distribution functions (CDFs), | specific weighting method | 8 fish sp | 1992-2006 | bts (EVHOE) | Bay of Biscay and Celtic Sea, North-East Atlantic |
| Reuchlin-Hugenholtz, Shackell and Hutchings (2015) | spatio-temporal dynamics | D90, G, area occupied, density area | by strata | 9 fish sp | 1970-2011 | bts | Scotian Shelf and Bay of Fundy |
| Rindorf, Lewy and Rose (2012) | spatio-temporal dynamics | Lloyds, I, Ellipses, Gini, D95, ... | not relevant | *Gadus morhue* |  |  | North sea |
| Rindorf and Lewy (2006) | spatio-temporal dynamics | CG (alternative formula) | not mentioned | *Gadus morhue* | 1983-2003 | bts | North Sea |
| Saraux et al. (2014) | spatio-temporal dynamics | *CG, I, IC, SP* | not mentioned | *Sardina pilchardus* and E*ngraulis encrasicolus* | 2003-2012 | as (PELMED) | Gulf of Lions |
| Spedicato, Woillez and Rivoirard (2007) | spatio-temporal dynamics | CG, I, i, IC, SP, PA, AS, EA, MS | mentioned but no detail given | *Mullus barbatus* | 1994-2004 | bts (GRUND/MEDITS) | **south Tyrrhenian sea** |
| Swain and Sinclair (1994) | spatio-temporal dynamics | G, D90 | not relevant | *Gadus morhue* |  |  | Gulf of St. Lawrence |
| Vikebø et al. (2005) | spatio-temporal dynamics | CG | not relevant |  |  |  |  |
| Volkenandt et al. (2014) | spatio-temporal dynamics | CG | mentioned but no detail given | *Clupea harengus* | 2005-2012 | as | Celtic sea |
| Woillez et al. (2007) | spatio-temporal dynamics | CG, I, i, IC, PA, SP, EA, MS, SP | mentioned but no detail given | *Merluccius merluccius* | 1987-2004 | bts | Bay of Biscay |
| Brodeur et al. (2014) | spatial overlap | CG, I, IC | not mentioned | *Clupea pallasii, engraulis mordax, Sardinops sagax* and *Chrysaora fuscescens* (medusae) | 1999-2011 | pelagic rope trawl | Washington and Oregon coast |
| Decker et al. (2018) | spatial overlap | CG, I, IC, Cramér-von Mises randomization test | not mentioned | *Gadus chalcogrammus*, *Clupea pallasii, Mallotus villosus* and *Gadus macrocephalus (fish)* and *Chrysaora melanaster* (jellyfish) | 2004-2012 | bts (NMFS AFSC and BASIS) | Bering Sea |
| Marino, Juanes and Stokesbury (2009) | spatial overlap | center of abundance, standard ellipse and 95%, confidence ellipse were Superimposed on the scallop density distribution maps to determine the spatial overlap | not relevant | *Placopecten magellanicus* (bivalve) | 1999-2006 | video surveys | Georges Bank |
| Tableau et al. (2016) | spatial overlap | CG, I, IC | not mentioned | 7 fish | 2008 | beam trawl (NURSE) | Bay of Vilaine (Bay of Biscay) |
| Petitgas et al. (2012) | growth | CG, I |  | *Engraulis encrasicolus* | 2001-2011 | fish otoliths | Bay of Biscay |
| Doray et al. (2017) | indicators integration | CG, I, i, PA, EA, MS | Voronoi with respective details | multiple (small pelagic fish) | 2000-2015 | as (PELGAS) | Bay of Biscay |
| Guan et al. (2017) | model based | CG, AO | not relevant (model based) | *Gadus morhue* | 1982-2013 | bts | Gulf of Maine |
| Thorson, Pinsky and Ward (2016) | model based | CG, AO | not relevant (model based) | 18 fish sp | 1977-2013 | bts | West coast of the USA |
| Thorson et al. (2016) | model based | CG, AO | not relevant (model based) | 92 sp of fish |  |  | 6 marine regions |
| Thorson (2017) | model based | CG, AO | not relevant (model based) | *Gadus chalcogrammus* | 1982-2017 | bts | Eastern Bering Sea |
| Chust et al. (2013) | model validation | CG | not relevant (model validation) | *Calanus* sp | 1959-2004 | Continuous Plankton Recorder survey coupled with model | North Atlantic Ocean |
| García-García, Ruiz-Villarreal and Bernal (2016) | model validation | CG, PA, EA, Coeffiicient of variation (PA/EA) | not relevant | *Sardina pilchardus* | 2006-2007 |  | Atlantic Iberian margin |
| Hinckley et al. (2016) | model validation | CG, I, i, Getis-Ord, NDI, Overlap coefficient, Syrjala tests | not relevant (model validation) | *Gadus chalcogrammus* |  |  | Gulf of Alasca |
| Huret, Petitgas and Woillez (Huret, Petitgas, and Woillez 2010) | model validation | CG, I, i, PA, AS, EA and coefficient of variation of positive values of densities | not relevant (model validation) | *Engraulis encrasicolus* (model of larvae dispersal) |  |  | Bay of Biscay |
| Lewy and Kristensen (Lewy and Kristensen 2009) | model validation | CG, concentration, AO | not relevant (model validation) | *Gadus morhue* | 1983-2006 | bts | North Sea |
| Petrik et al. (2015) | model validation | CG, I, IC | not relevant (model validation) |  |  |  |  |
| Pointin et al. (2018) | model validation | CG, I, IC | not mentioned | discards/landings | 2011-2016 | onboard observers (OBSMER) | Celtic Sea and western English Channel |

**References:**

Adams, Charles F. 2017. “Age-Specific Differences in the Seasonal Spatial Distribution of Butterfish (*Peprilus Triacanthus*).” *ICES Journal of Marine Science* 74(1):170–79.

Adams, Charles F. et al. 2018. “Relative Importance of Population Size, Fishing Pressure and Temperature on the Spatial Distribution of Nine Northwest Atlantic Groundfish Stocks” edited by G. Ottersen. *PLOS ONE* 13(4):e0196583. Retrieved May 29, 2018 (http://dx.plos.org/10.1371/journal.pone.0196583).

Alvarez, P., L. Moros, A. Uriarte, and J. Egaña. 2001. “Spatial and Temporal Distribution of European Hake, *Merluccius Merluccius* (L.), Eggs and Larvae in Relation to Hydrographical Conditions in the Bay of Biscay.” *Fisheries Research* 50(1–2):111–28.

Alvarez, P., L. Moros, A. Uriarte, and J. Egaña. 2004. “Distribution and Abundance of European Hake *Merluccius Merluccius* Eggs and Larvae in the North East Atlantic Waters in 1995 and 1998 in Relation to Hydrographic Conditions.” *Journal of Plankton Research* 26(7):811–26.

Atkinson, D. B., G. A. Rose, E. F. Murphy, and C. A. Bishop. 1997. “Distribution Changes and Abundance of Northern Cod (Gadus Morhua), 1981–1993.” *Canadian Journal of Fisheries and Aquatic Sciences* 54(3):132–38. Retrieved (http://www.nrc.ca/cgi-bin/cisti/journals/rp/rp2_abst_e?cjfas_f96-158_54_ns_nf_cjfas54-97).

Barra, Marco et al. 2015. “Interannual Changes in Biomass Affect the Spatial Aggregations of Anchovy and Sardine as Evidenced by Geostatistical and Spatial Indicators.” *PLoS ONE* 10(8). Retrieved March 30, 2018 (http://journals.plos.org/plosone/article?id=10.1371/journal.pone.0135808).

Baudron, Alan R. and Paul G. Fernandes. 2015. “Adverse Consequences of Stock Recovery: European Hake, a New ‘Choke’ Species under a Discard Ban?” *Fish and Fisheries* 16(4):563–75. Retrieved March 30, 2018 (http://doi.wiley.com/10.1111/faf.12079).

Bez, Nicolas and Jacques Rivoirard. 2000. “On the Role of Sea Surface Temperature on the Spatial Distribution of Early Stages of Mackerel Using Inertiograms.” *ICES Journal of Marine Science* 57(2):383–92. Retrieved (Bez & Rivoirard2000.pdf).

Bitetto, I., M. T. Facchini, M. T. Spedicato, and G. Lembo. 2012. “Spatial Location of Giant Red Shrimp (*Aristaeomorpha Foliacea*, Risso , 1827) in the Central-Southern Tyrrhenian Sea.” *Biologia Marina Mediterranea* 19(1):92–95. Retrieved March 30, 2018 (https://www.researchgate.net/profile/Isabella_Bitetto/publication/260255663_Spatial_location_of_giant_red_shrimp_Aristaeomorpha_foliacea_Risso1827_in_the_central-southern_Tyrrhenian_Sea/links/00b495305c29370b9c000000/Spatial-location-of-giant-red-shrimp-A).

Blanchard, Julia L. et al. 2005. “Distribution–Abundance Relationships for North Sea Atlantic Cod (*Gadus Morhua*): Observation versus Theory.” *Canadian Journal of Fisheries and Aquatic Sciences* 62(9):2001–9. Retrieved (http://www.nrcresearchpress.com/doi/abs/10.1139/f05-109).

Bonanno, A. et al. 2017. “Space Utilization by Key Species of the Pelagic Fish Community in an Upwelling Ecosystem of the Mediterranean Sea.” *Hydrobiologia*, August 19, 1–18. Retrieved March 30, 2018 (http://link.springer.com/10.1007/s10750-017-3350-9).

Bourdaud, Pierre, Morgane Travers-Trolet, Youen Vermard, Xochitl Cormon, and Paul Marchal. 2017. “Inferring the Annual, Seasonal, and Spatial Distributions of Marine Species from Complementary Research and Commercial Vessels’ Catch Rates.” *ICES Journal of Marine Science* 74(9):2415–26. Retrieved March 30, 2018 (https://academic.oup.com/icesjms/article-abstract/74/9/2415/3858384).

Boyra, G. et al. 2013. “Acoustic Surveys for Juvenile Anchovy in the Bay of Biscay: Abundance Estimate as an Indicator of the next Year’s Recruitment and Spatial Distribution Patterns.” *ICES Journal of Marine Science* 70(7):1354–68. Retrieved March 30, 2018 (https://academic.oup.com/icesjms/article-abstract/70/7/1354/608431).

Brodeur, Richard D., Caren Barceló, Kelly L. Robinson, Elizabeth A. Daly, and James J. Ruzicka. 2014. “Spatial Overlap between Forage Fishes and the Large Medusa *Chrysaora Fuscescens* in the Northern California Current Region.” *Marine Ecology Progpelress Series* 510:167–81. Retrieved March 30, 2018 (http://www.int-res.com/abstracts/meps/v510/p167-181/).

Bruge, Antoine, Paula Alvarez, Almudena Fontán, Unai Cotano, and Guillem Chust. 2016. “Thermal Niche Tracking and Future Distribution of Atlantic Mackerel Spawning in Response to Ocean Warming.” *Frontiers in Marine Science* 3. Retrieved March 30, 2018 (https://www.frontiersin.org/article/10.3389/fmars.2016.00086).

Chust, Guillem et al. 2013. “Are *Calanus* Sp. Shifting Poleward in the North Atlantic? A Habitat Modelling Approach.” *ICES Journal of Marine Science*. Retrieved March 30, 2018 (https://academic.oup.com/icesjms/article-abstract/71/2/241/787087).

Decker, Mary Beth et al. 2018. “Jellyfish and Forage Fish Spatial Overlap on the Eastern Bering Sea Shelf during Periods of High and Low Jellyfish Biomass.” *Marine Ecology Progress Series* 591:57–69. Retrieved March 30, 2018 (http://www.int-res.com/abstracts/meps/v591/p57-69/).

Doray, Mathieu et al. 2017. “Monitoring Small Pelagic Fish in the Bay of Biscay Ecosystem, Using Indicators from an Integrated Survey.” *Progress in Oceanography* (xxxx):0–1. Retrieved (http://dx.doi.org/10.1016/j.pocean.2017.12.004).

Engelhard, Georg H., John K. Pinnegar, Laurence T. Kell, and Adriaan D. Rijnsdorp. 2011. “Nine Decades of North Sea Sole and Plaice Distribution.” *ICES Journal of Marine Science* 68(6):1090–1104.

Faraj, Abdelmalek and Nicolas Bez. 2007. “Spatial Considerations for the Dakhla Stock of *Octopus Vulgaris*: Indicators, Patterns, and Fisheries Interactions.” *ICES Journal of Marine Science* 64(9):1820–28.

García-García, Luz María, Manuel Ruiz-Villarreal, and Miguel Bernal. 2016. “A Biophysical Model for Simulating Early Life Stages of Sardine in the Iberian Atlantic Stock.” *Fisheries Research* 173:250–72. Retrieved March 30, 2018 (https://www.sciencedirect.com/science/article/pii/S0165783615300990).

Gastauer, Sven et al. 2016. “The Distribution of Blue Whiting West of the British Isles and Ireland.” *Fisheries Research* 183:32–43. Retrieved March 30, 2018 (https://www.sciencedirect.com/science/article/pii/S0165783616301497).

Guan, Lisha et al. 2017. “The Influence of Complex Structure on the Spatial Dynamics of Atlantic Cod (*Gadus Morhua*) in the Gulf of Maine.” *ICES Journal of Marine Science* 74(9):2379–88. Retrieved March 30, 2018 (https://academic.oup.com/icesjms/article-abstract/74/9/2379/3769381).

Hinckley, Sarah, Carolina Parada, John K. Horne, Michael Mazur, and Mathieu Woillez. 2016. “Comparison of Individual-Based Model Output to Data Using a Model of Walleye Pollock Early Life History in the Gulf of Alaska.” *Deep-Sea Research II* 132:240–62. Retrieved May 25, 2018 (https://ac.els-cdn.com/S0967064516300790/1-s2.0-S0967064516300790-main.pdf?_tid=fe6ec701-d097-47b1-a323-82aa4423b568&acdnat=1527264282_3dd782a2ac922040cff35160ce5fd112).

Honkalehto, Taina, Patrick H. Ressler, Richard H. Towler, Christopher D. Wilson, and Josef Michael Jech. 2011. “Using Acoustic Data from Fishing Vessels to Estimate Walleye Pollock (Theragra Chalcogramma) Abundance in the Eastern Bering Sea” edited by J. M. Jech. *Canadian Journal of Fisheries and Aquatic Sciences* 68(7):1231–42. Retrieved March 30, 2018 (http://www.nrcresearchpress.com/doi/abs/10.1139/f2011-050).

Hughes, Kathryn M., Leonie Dransfeld, and Mark P. Johnson. 2014. “Changes in the Spatial Distribution of Spawning Activity by North-East Atlantic Mackerel in Warming Seas: 1977–2010.” *Marine Biology* 161(11):2563–76.

Huret, M., P. Petitgas, and M. Woillez. 2010. “Dispersal Kernels and Their Drivers Captured with a Hydrodynamic Model and Spatial Indices: A Case Study on Anchovy (*Engraulis Encrasicolus*) Early Life Stages in the Bay of Biscay.” *Progress in Oceanography* 87(1–4):6–17. Retrieved (http://dx.doi.org/10.1016/j.pocean.2010.09.023).

Lewy, Peter and Kasper Kristensen. 2009. “Modelling the Distribution of Fish Accounting for Spatial Correlation and Overdispersion.” *Canadian Journal of Fisheries and Aquatic Sciences* 66(10):1809–20. Retrieved May 29, 2018 (http://www.nrcresearchpress.com/doi/pdf/10.1139/F09-114).

Marino, Michael C., Francis Juanes, and Kevin D. E. Stokesbury. 2009. “Spatio-Temporal Variations of Sea Star *Asterias* Spp. Distributions between Sea Scallop *Placopecten Magellanicus* Beds on Georges Bank.” *Marine Ecology Progress Series* 382:59–68.

Modica, Larissa, Pilar Córdoba, Cristina Rodríguez-Cabello, Francisco Sánchez, and Francisco Velasco. 2016. “A New Approach to Species Distributional Indicators for the Marine Strategy Framework Directive (MSFD).” *Ecological Indicators* 67:21–30. Retrieved March 30, 2018 (https://www.sciencedirect.com/science/article/pii/S1470160X16300255).

Morfin, Marie, Jean-Marc Fromentin, Angélique Jadaud, and Nicolas Bez. 2012. “Spatio-Temporal Patterns of Key Exploited Marine Species in the Northwestern Mediterranean Sea.” *PLoS ONE* 7(5):e37907.

Murawski, S. I. A., J. T. Finn, and I. T. Finn. 1988. “Biological Bases for Mixed-Species Fisheries: Species Co-Distribution in Relation to Environmental and Biotic Variables.” *Canadian Journal of Fisheries and Aquatic Sciences* 45(10):1720–35. Retrieved (http://dx.doi.org/10.1139/f88-204).

Myers, R. A., B. Brodie, N. J. Barrowman, and R. Bowering. 1995. *Changes in the Concentration of Flatfish off Newfoundland from 1971 to 1994*.

Paulino, Carlos, Eloy Aroni, Han Xu, Edward Alburqueque, and Hervé Demarcq. 2017. “Use of Nighttime Visible Images in the Study of the Spatial and Temporal Variability of Fishing Areas of Jumbo Flying Squid (*Dosidicus Gigas*) Outside Peruvian EEZ 2004–2015.” *Fisheries Research* 191:144–53. Retrieved March 30, 2018 (https://www.sciencedirect.com/science/article/pii/S0165783617300760).

Perry, Allison L., Paula J. Low, Jim R. Ellis, and John D. Reynolds. 2014. “Climate Change and Distribution Shifts in Marine Fishes.” *Science* 308(5730):1912–15. Retrieved April 10, 2017 (http://science.sciencemag.org/content/308/5730/1912.full).

Persohn, Cecile, Pascal Lorance, and Verena M. Trenkel. 2009. “Habitat Preferences of Selected Demersal Fish Species in the Bay of Biscay and Celtic Sea, North-East Atlantic.” *Fisheries Oceanography* 18(4):268–85.

Petitgas, P., P. Grellier, … E. Duhamel-ICES Annual science, and Undefined 2012. 2012. “Variability and Controls of Otolith Growth in the Anchovy of the Bay of Biscay.” *Archimer.Ifremer.Fr* J:18. Retrieved March 30, 2018 (http://archimer.ifremer.fr/doc/00114/22550/).

Petitgas, Pierre and Jean-Charles Poulard. 2009. “A Multivariate Indicator to Monitor Changes in Spatial Patterns of Age-Structured Fish Populations.” *Aquatic Living Resources* 22(2):165–71. Retrieved March 30, 2018 (https://www.cambridge.org/core/journals/aquatic-living-resources/article/multivariate-indicator-to-monitor-changes-in-spatial-patterns-of-agestructured-fish-populations/4FE49D9F78F7E1C2964F9D6971C013A7).

Petrik, Colleen M., Janet T. Duffy-Anderson, Franz Mueter, Katherine Hedstrom, and Enrique N. Curchitser. 2015. “Biophysical Transport Model Suggests Climate Variability Determines Distribution of Walleye Pollock Early Life Stages in the Eastern Bering Sea through Effects on Spawning.” *Progress in Oceanography* 138:459–74. Retrieved (http://dx.doi.org/10.1016/j.pocean.2014.06.004).

Pointin, Fabien, Anne Sophie Cornou, Romain Prod’homme, Nicolas Taupin, and Marie Joëlle Rochet. 2018. “A Method to Address the Non-Random Spatial Distribution of on-Board Observer Data to Map Landings and Discards.” *Fisheries Research* 199:242–51. Retrieved March 30, 2018 (https://www.sciencedirect.com/science/article/pii/S0165783617302989).

Reuchlin-Hugenholtz, Emilie, Nancy L. Shackell, and Jeffrey A. Hutchings. 2015. “The Potential for Spatial Distribution Indices to Signal Thresholds in Marine Fish Biomass” edited by K. I. Stergiou. *PLoS ONE* 10(3):e0120500. Retrieved March 30, 2018 (http://dx.plos.org/10.1371/journal.pone.0120500).

Rindorf, A. and P. Lewy. 2006. “Warm, Windy Winters Drive Cod North and Homing of Spawners Keeps Them There.” *Journal of Animal Ecology* 43:445–53.

Rindorf, Anna, Peter Lewy, and Kenneth A. Rose. 2012. “Estimating the Relationship between Abundance and Distribution.” *Canadian Journal of Fisheries and Aquatic Sciences* 69(2):382–97.

De Robertis, Alex and Edward D. Cokelet. 2012. “Distribution of Fish and Macrozooplankton in Ice-Covered and Open-Water Areas of the Eastern Bering Sea.” *Deep Sea Research Part II: Topical Studies in Oceanography* 65–70:217–29. Retrieved May 31, 2018 (https://www.sciencedirect.com/science/article/pii/S0967064512000094#f0010).

Saraux, Claire et al. 2014. “Spatial Structure and Distribution of Small Pelagic Fish in the Northwestern Mediterranean Sea.” *PLoS ONE* 9(11):e111211.

Spedicato, Mt, Mathieu Woillez, and Jacques Rivoirard. 2007. “Usefulness of the Spatial Indices to Define the Distribution Pattern of Key Life Stages: An Application to the Red Mullet (*Mullus Barbatus*) Population in the South Tyrrhenian Sea.” *Ices CM* O:10:1–18. Retrieved (http://info.ices.dk/products/AnnualRep/ASCproceedings/2007/Annual Science Conference 2007/CM-2007/O/O1007.pdf).

Swain, D. P. and a. F. Sinclair. 1994. “Fish Distribution and Catchability: What Is the Appropriate Measure of Distribution?” *Canadian Journal of Fisheries and Aquatic Sciences* 51(5):1046–54. Retrieved (http://dx.doi.org/10.1139/f94-104).

Tableau, A., A. Brind’Amour, M. Woillez, and H. Le Bris. 2016. “Influence of Food Availability on the Spatial Distribution of Juvenile Fish within Soft Sediment Nursery Habitats.” *Journal of Sea Research* 111:76–87.

Thorson, James T. 2017. “The Relative Influence of Temperature and Size Structure on Fish Distribution Shifts: A Case Study on Walleye Pollock in the Bering Sea.” *Fish and Fisheries* 1–12.

Thorson, James T., Malin L. Pinsky, and Eric J. Ward. 2016. “Model-Based Inference for Estimating Shifts in Species Distribution, Area Occupied and Centre of Gravity” edited by O. Gimenez. *Methods in Ecology and Evolution* 7(8):990–1002. Retrieved April 5, 2017 (http://doi.wiley.com/10.1111/2041-210X.12567).

Thorson, James T., Anna Rindorf, Jin Gao, Dana Hanselman, and Henning Winker. 2016. “Density-Dependent Changes in Effective Area Occupied for Sea-Bottom-Associated Marine Fishes.” *Philosophical Transactions of the Royal Society B: Biological Sciences* 283(1840):20161853. Retrieved April 5, 2017 (http://rspb.royalsocietypublishing.org/content/283/1840/20161853).

Vikebø, Frode, Svein Sundby, Bjørn Ådlandsvik, and Øyvind Fiksen. 2005. “The Combined Effect of Transport and Temperature on Distribution and Growth of Larvae and Pelagic Juveniles of Arcto-Norwegian Cod.” *ICES Journal of Marine Science* 62(7):1375–86.

Volkenandt, Mareike, Simon Berrow, Ian O’Connor, Jean Marc Guarini, and Ciaran O’Donnell. 2014. “Prespawning Herring Distribution in the Irish Celtic Sea between 2005 and 2012.” *ICES Journal of Marine Science* 72(2):498–507. Retrieved March 30, 2018 (https://academic.oup.com/icesjms/article-abstract/72/2/498/2801379).

Woillez, Mathieu, Jean-Charles Poulard, Jacques Rivoirard, Pierre Petitgas, and Nicolas Bez. 2007. “Indices for Capturing Spatial Patterns and Their Evolution in Time, with Application to European Hake (Merluccius Merluccius) in the Bay of Biscay.” *ICES Journal of Marine Science:* 64(3):537–50.

Woillez, Mathieu, Jacques Rivoirard, and Pierre Petitgas. 2009. “Notes on Survey-Based Spatial Indicators for Monitoring Fish Populations.” *Aquatic Living Resources* 22(2):155–64.
