# Supplementary material for: Integrating spatial indicators in the surveillance of exploited marine ecosystems
Source: PLoS One. 2018 Nov 21;13(11):e0207538. doi: 10.1371/journal.pone.0207538 (PMC6248972; doi:10.1371/journal.pone.0207538)

##### S2: Illustration of spatial indicators

Illustration of some spatial indicators mentioned in the text: Gini index, Lorenz curve and spreading area (upper panel), center of gravity, inertia and isotropy (lower panel).


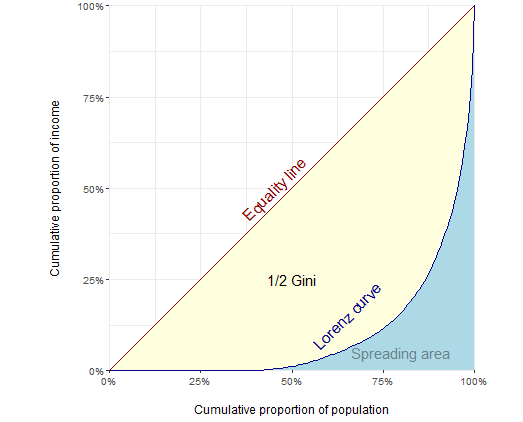


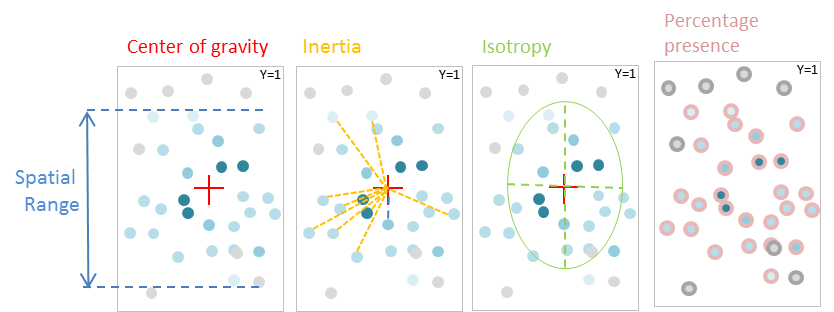

Supplement: S2 File — Illustration of some spatial indicators mentioned in the text: Gini index, Lorenz curve and spreading area (upper panel), center of gravity, inertia and isotropy (lower panel). (DOCX) [file pone.0207538.s002.docx]
