# Supplementary material for: Integrating spatial indicators in the surveillance of exploited marine ecosystems
Source: PLoS One. 2018 Nov 21;13(11):e0207538. doi: 10.1371/journal.pone.0207538 (PMC6248972; doi:10.1371/journal.pone.0207538)

### S3: Options on the estimation of the areas of influence

Six of the indicators under study were computed with and without weighting by their areas of influence. In relation to the two parameters required to calculate the areas of influence using Voronoï, the results show that no matter the species, year, or indicator there was always a clear plateau reached, in relation to the dmax and the number of nodes required for the Voronoï. This suggests that the two parameters can be determined easily prior to the indicator analysis. The results given by the six indicators estimated considering constant areas of influence around each sample (S) or variable areas of influence estimated using Voronoï were highly correlated notwithstanding the species distributions. Indeed, differences in the scales of the indicators were observed, most likely induced by the weighting factor, but the coefficients of determination ranged between 0.66 to 0.96. For the coordinates of the center of gravity (CGlat and CGlong), the inertia and isotropy (iso), there was no change in the relationship with the surveys, whereas for the index of aggregation (Iagg), positive area (parea), spreading area (sparea) and equivalent area (eqarea) the relationship changed strongly with the studied survey, suggesting an effect of the sampling design (transects *vs* randomly stratified).

Representation of different options on the estimation of the areas of influence, using RGeostats.


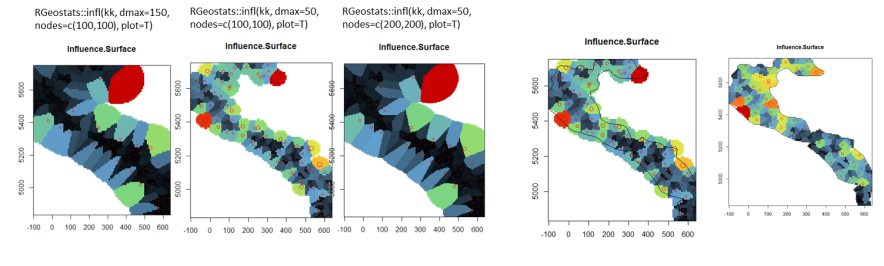


Effect of increasing DMAX parameter, in the weighted indicator’s result, estimated using *Zeus faber* annual distributions (nodes=c(200,200)). Note a clear plateau is reached after dmax=100.


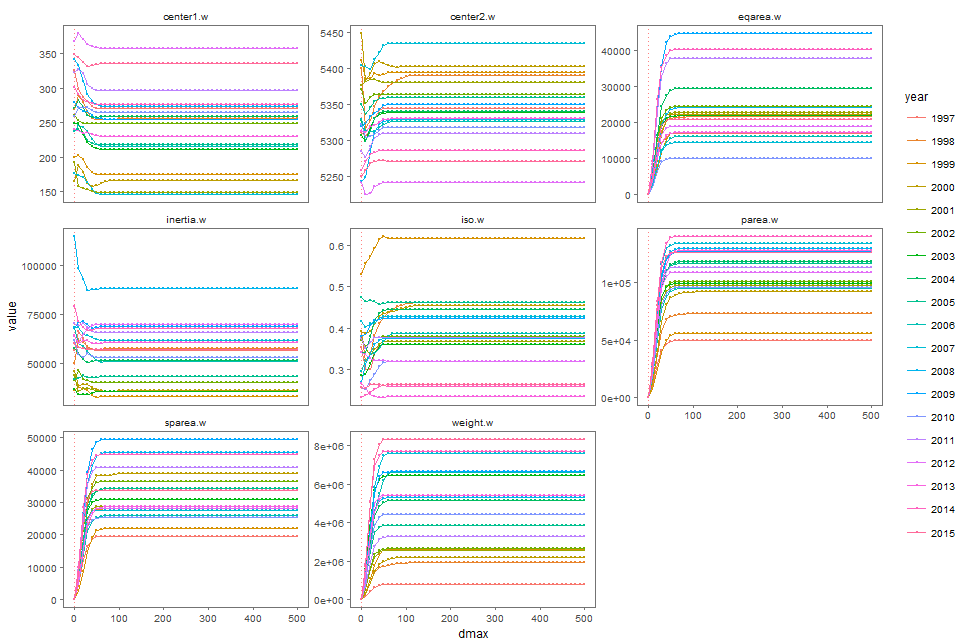


Effect of progressive increase of the parameter ‘maximum distance between data and target’ (dmax), in the final weighted indicators result, for different sampled species, during 2015 (nodes=c(200,200)). Note a clear plateau is reached after dmax=100.


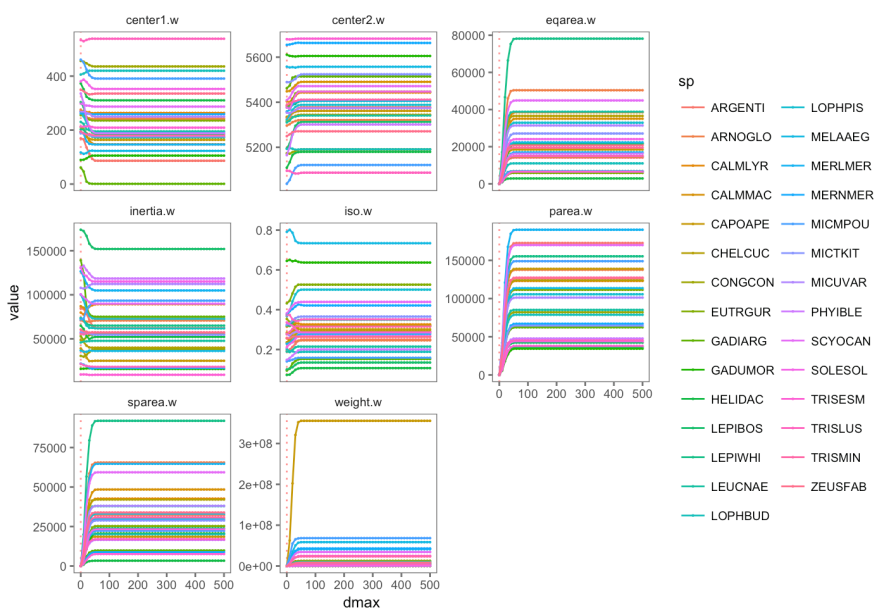


Effect of progressive increase of the ‘number of nodes of the discretization grid’ parameter (node), in the weighted indicators result, for different years of *Zeus faber* distribution (dmax=100). Note a clear plateau is reached after dmax=100.


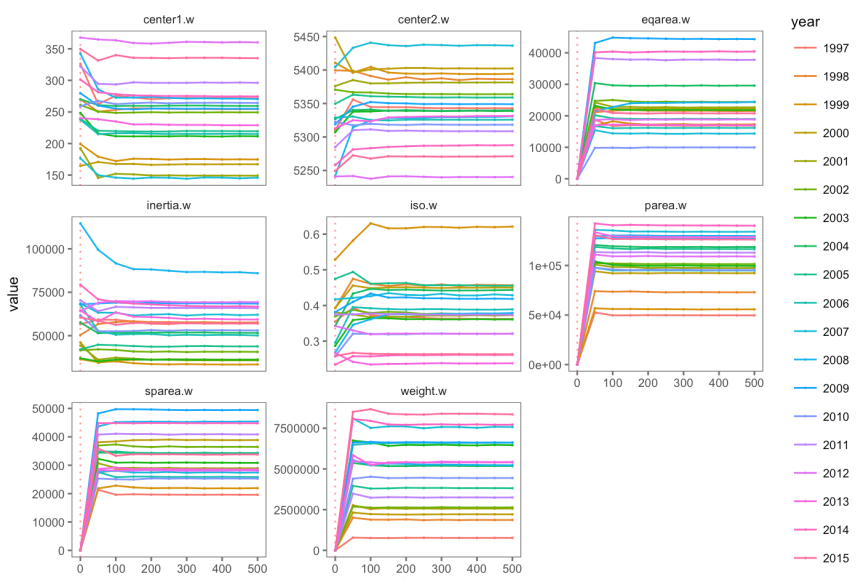


Effect of progressive increase of node parameter, in the weighted indicators result, for different sampled species, during 2015 (dmax=100). Note a clear plateau is reached after dmax=100.


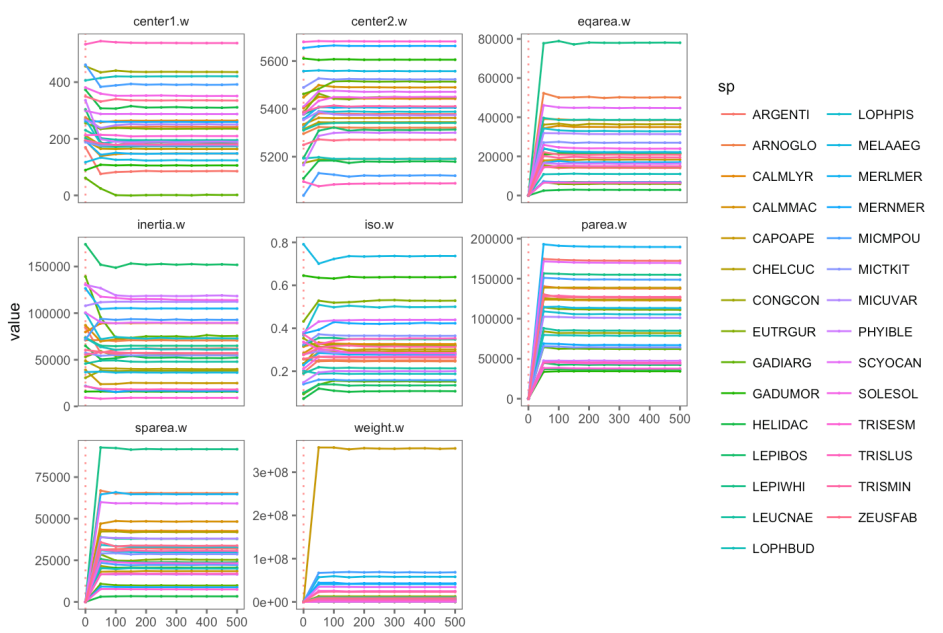


Effect of incorporating the areas of influence on the indicators estimation. Relationship between the indicators estimated using raw data (S. prefix) and the respective indicators estimated taking into account the surface areas estimated by Voronoï (W. prefix), with the respective regression line and R2. Blue circles indicate the pelagic survey species distribution, whereas orange triangles, the bottom trawl survey. Accordingly, when there was no difference in the relationship by survey, a global model was estimated, whereas when the relationship differed according to survey, the model shown was estimated by survey.


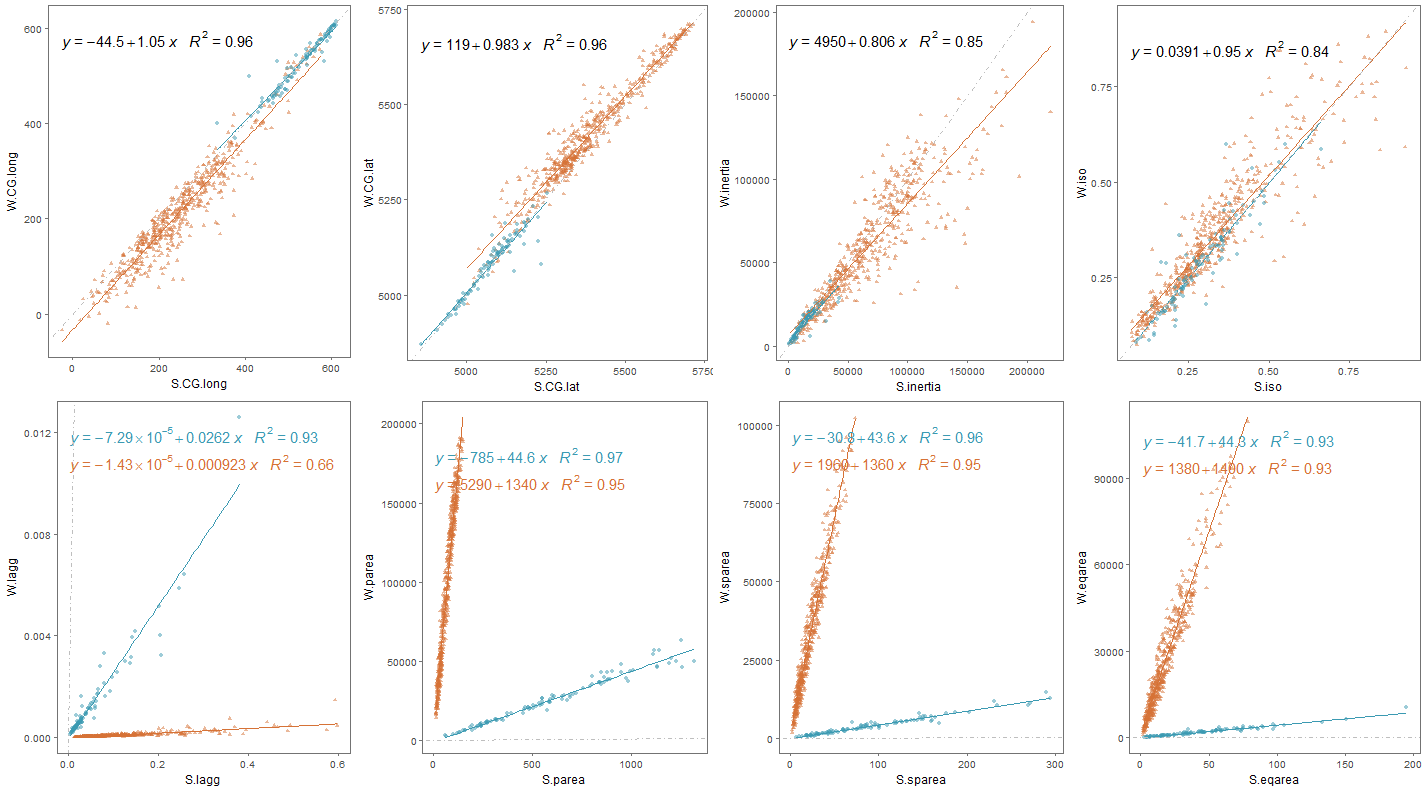

Supplement: S3 File — (DOCX) [file pone.0207538.s003.docx]
