# Supplementary figures and images for: Integrating spatial indicators in the surveillance of exploited marine ecosystems
Source: PLoS One. 2018 Nov 21;13(11):e0207538. doi: 10.1371/journal.pone.0207538 (PMC6248972; doi:10.1371/journal.pone.0207538)

# S4: PCA with complete matrix (species and years)


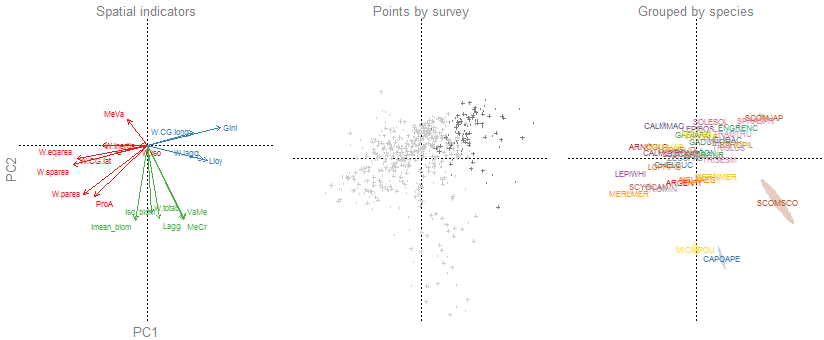

Supplement: S4 File — (DOCX) [file pone.0207538.s004.docx]
